# Functional specialisation across the first five years of life: A longitudinal characterisation of social perception with fNIRS

**DOI:** 10.1162/IMAG.a.1368

**Published:** 2026-09-17

**Authors:** Johann Benerradi, Chiara Bulgarelli, Ebrima Mbye, Borja Blanco, Ebou Touray, Sam McCann, Bosiljka Milosavljevic, Sophie E. Moore, Clare E. Elwell, Anna Blasi, Sarah Lloyd-Fox

**Affiliations:** University of Cambridge, Cambridge, United Kingdom; Birkbeck University of London, London, United Kingdom; MRC Unit The Gambia at the London School of Hygiene and Tropical Medicine, Banjul, The Gambia; King’s College London, London, United Kingdom; Queen Mary University, London, United Kingdom; University College London, London, United Kingdom

**Keywords:** fNIRS, neurodevelopment, social, longitudinal, infancy, toddlerhood

## Abstract

Research in typically developing infants has shown robust and consistent brain activation to social versus non-social visual and auditory stimuli in a network of brain regions, including the inferior frontal, anterior temporal, and posterior superior temporal cortex. However, large-scale, longitudinal neuroimaging studies across early childhood, particularly in low- and middle-income countries, are rare, yet important, given that they offer a powerful means of capturing within-person changes in neurodevelopment and identifying targets for intervention and support. Here, we investigated brain responses to social perception longitudinally across the first 5 years of life of participants in The Gambia with functional near-infrared spectroscopy (fNIRS) within the Brain Imaging for Global Health (BRIGHT) Project. We found that social visual stimuli elicited a selective right posterior temporal response across all six age points studied here (5, 8, 12, 18, 24 months and 3–5 years of age), with concurrent selective left responses at four of the six time points (5, 8, 18 months and 3–5 years of age). Inferior frontal regions showed brain activation at multiple ages, with the youngest (5 months) and oldest (24 months to 3–5 years) time points evidencing more widespread frontal social visual responses. Conversely, auditory social stimuli elicited a significantly stronger and more prolonged response than auditory non-social stimuli across all six age points from 5 months to 3–5 years across a range of frontal and temporal areas. Finally, from individual infant trajectories of brain activation, we identified different profiles of age-dependent specialization to auditory social stimuli, with some specializing earlier than others. Future work could take advantage of such profiles to investigate the effects of adversity and resilience factors on neurodevelopment.

## 1 Introduction

Nearly 1 billion children worldwide are growing up in poverty, facing challenges that impact lifelong health and development (“Child Poverty.” UNICEF. https://www.unicef.org/social-policy/child-poverty). These environments, characterized by limited access to essential resources such as adequate nutrition, healthcare, and education, expose infants to various genetic, environmental, and psychosocial risks that can have lasting effects on their cognitive and physical development. Exposure to risks such as chronic stress, malnutrition, and inadequate stimulation can disrupt a child’s healthy neural development and affect early developmental trajectories in language and cognition (Jensen et al., 2017, 2019). Indeed, one-third of pre-school-aged children in developing countries fail to reach their developmental milestones in cognitive or socioemotional growth, with the largest number of affected children in sub-Saharan Africa (McCoy et al., 2016). Not only can this lead to deficits in the acquisition of key skills such as delays in attention and communication milestones, but it can also lead to poorer academic achievements and a lasting impact into adulthood.

The first 1,000 days of life, between conception and 2 years of age, are characterized by rapid physical, physiological, and psychological development. Investigating development from the earliest stages of life is essential for identifying potential deficits and informing the design of targeted, developmentally appropriate interventions, where needed. Infants’ brain plasticity and specialization in response to environmental factors plays a critical role in early development. Mapping brain function across the first 2 years of life is key to understanding the ages at which detrimental effects appear, and the contextual factors most closely associated with subsequent neural or cognitive differences up to 5 years when children reach school age.

A key area of early development is how infants respond, interpret, and learn from their social world. The capacity to engage and communicate in a social world is one of the defining characteristics of the human species. Given that human infants are reliant on their caregivers for an extensive period of their development, opportunities for early interactions with their social environment are largely driven by their caregivers. Therefore, risk factors that disrupt caregivers’ lives (i.e., unstable family income, food insecurity, mental health issues, life stressors, climate impacts) will also impact their ability to provide sensitive, responsive, and extensive opportunities for social interaction, which, in turn, may impact early neural trajectories of our capacity to perceive differing social cues. The link between life stressors, early adversity, socioemotional development, and social cognition has been widely studied over the last century. Previous research following the Great Depression of the United States of America found that economic loss led to changes in the quality of caregiving which, in turn, associated with early socioemotional development (Elder, 1979). Grantham-McGregor and colleagues (Grantham-McGregor et al., 1991) investigated the effects of nutritional supplementation and psychosocial stimulation for infants living in low-resource contexts in Jamaica, finding that those infants with caregivers who learnt more sensitive caregiving practices and social interaction skills had enhanced cognitive development at pre-school age, and positive benefits extending into adulthood (Walker et al., 2022). Furthermore, predictive modeling across 35 low- and middle-income countries (LMICs) of the Early Childhood Development Index (ECDI) has shown that socio-emotional development of pre-schoolers (3–4 years) is impacted more extensively (26.2% of cohort with low scores) than cognitive development (14.6%) by associated risk factors (McCoy et al., 2016).

Recent efforts to understand the neural mechanisms by which early social cognition and socioemotional development are impacted by adversity and risk factors are limited but provide some insight. In a pilot work conducted in The Gambia using functional near-infrared spectroscopy (fNIRS) to study social perception neural responses, we found evidence of age-related changes in visual- and auditory-social selectivity across the first 2 years of life (Lloyd-Fox et al., 2013, 2017). However, this research had some limitations: (i) while the study was based in a low-resource context, it did not directly study associations with adversity factors due to the low sample size; (ii) the study investigated responses at 1 and 24 months cross-sectionally, or with a more limited longitudinal sample from 9–16 months; and finally, (iii) the neuroimaging headgear was a small feasibility kit that only covered one region of interest in the right hemisphere making the investigation of broader social network activation impossible. On the other hand, a study in Bangladesh has shown that brain activation to social compared to non-social stimuli in 6-, 24-, and 36-month-olds was related to poverty, maternal education, maternal stress, caregiver violence, and verbal abuse (Perdue et al., 2019; Pirazzoli et al., 2022). While this study contributed considerably to our understanding of the neural mechanisms of social cognition and associations with adverse contextual factors, it studied each participant at only 1–2 time points, with the 6-24-month and 36-month cohorts being separate. An objective of the work presented herewith will therefore be to investigate changes in social selectivity with a larger individual age span than previous work to validate findings which described a switch from non-vocal selectivity to vocal selectivity during the first year of life (Grossmann et al., 2010; Lloyd-Fox et al., 2012, 2017).

Conducting longitudinal studies across an extensive early developmental window (e.g., from birth to school age) is essential, as it allows researchers not only to identify when individual skills and neural markers emerge, but also to map the trajectories of cognitive development over time. When combined with other contextual measures, this approach is important for investigating the factors that influence developmental trends and for identifying distinct developmental profiles. Longitudinal studies offer a much larger window to capture age-related changes that may develop rapidly at key developmental stages, while also enabling a clearer understanding of causal relationships.

However, longitudinal neuroimaging studies are scarce (Azhari et al., 2020), and very few neurodevelopmental studies have been conducted in LMICs (Blasi et al., 2019), with literature identified from just two contexts: Bangladesh (Perdue et al., 2019) and India (Wijeakumar et al., 2019). The scarcity of neuroimaging studies in LMICs is partly due to the typically high cost of neuroimaging equipment and shortage of well-resourced research labs (which until recently was the only context in which neuroimaging research studies were routinely undertaken). However, recently, the adoption of new more affordable, flexible, and easy-to-use neuroimaging technologies such as fNIRS and electroencephalography (EEG) in LMICs has made it possible to measure functional brain responses in more varied contexts (Blasi et al., 2019; Gervain et al., 2023). In 2014, the interdisciplinary, longitudinal Brain Imaging for Global Health (BRIGHT) Project was launched with the aim to expand diversity in neurodevelopmental studies and establish brain function-for-age curves in infants from high- (UK) and low-resource (The Gambia) settings in order to gain an insight into the effects that contextual factors related to living in a low-resource context may have on infant development (Lloyd-Fox et al., 2024). To achieve this aim, a wide range of measures were employed, including neuroimaging (fNIRS and EEG), eye tracking, and physiological measures, alongside behavioral and cognitive assessments and measures of quality of the child’s environment and caregiving practices. These were complemented by a wealth of data on both biological (physical size and biomarkers of nutritional status and inflammation) and psychosocial risk factors (e.g., questionnaire-based measures of adversity exposure and parental mental health)).

To investigate the developmental trajectory of our capacity to perceive social cues across early childhood, the BRIGHT Project utilized a social perception fNIRS task to capture visual and auditory social neural responses from birth to 5 years of age. To our knowledge, this is the first neuroimaging study to measure developmental longitudinal neural trajectories of social perception across this number of time points, with this extensive early childhood age range and with this sample size. Therefore, while in future work we aim to include these data in modeling pathways to investigate the impact of adversity, a first priority was to better understand the origin, specialization patterns, and developmental trajectories of visual and auditory social perception and isolate potential biomarkers.

Due to the sample size and number of time points, this work is also motivated by the aim of better understanding whether methodological and analytical parameters may impact accurate profiling of developmental trajectories. One motivation to explore individual differences further is that previous literature indicates that when comparing speech, non-speech human vocalizations, and non-human biological or non-biological sounds, infants display evidence of developmental specialization to vocal stimuli across the first year of life (Grossmann et al., 2010; Lloyd-Fox et al., 2012, 2017; Minagawa-Kawai et al., 2011; Shultz et al., 2014). While this has been validated through several replication studies, in different populations and with the use of varying stimuli, some differences have arisen. This may suggest considerable individual infant variability of specialisation, which may be related to contextual factors of interest. However, it is possible that the reported significant findings may also vary for methodological reasons such as:

- which brain region was investigated (i.e., temporal only, left hemisphere vs. bilateral, several social or language network regions);
- how the analyses were conducted (i.e., selection of a specific time windows of interest post-stimulus onset used for feature extraction, pre-defined regions of interest from previous literature, hardware constraints on extent of the anatomical region that can be investigated);
- which stimulus choice of comparison was used (i.e., speech vs. communicative vocal sounds (yawn or laugh) vs. non-speech biological sounds (walking), or vocal (coughing, crying, laughing or yawning) vs. non-vocal sounds (water or toys));
- which part of the hemodynamic signal was focused on (i.e., for oxyhaemoglobin (HbO) only or both HbO and deoxyhaemoglobin (HbR), for stimulus comparison, focusing on optimal parameters to capture stimulus of interest rather than baseline);
- which age point the study focused on (i.e., studies tend to be cross-sectional or focus on a narrow age span such as 1 to 4 months or 4 to 7 months).

The larger sample size and the wide longitudinal age range including multiple testing sessions used in the BRIGHT project can allow us to investigate some of these factors. Firstly, we can research age-related changes more comprehensively. Secondly, we can investigate whether the analytical choices bias findings to be significant for the stimulus of interest (i.e., an analytical time window optimized for the social response may undervalue the response seen to the contrasting auditory non-social stimuli). Thirdly, we can explore whether notable age-related group level and individual differences may arise if we widen our scope of potential biomarkers to include a range of hemodynamic response characteristics. These include the standard approach to identify the localized peak response within a given region using an average hemodynamic change analysis, as well as investigating time-to-peak (latency), magnitude of response to stimulus relative to baseline, and spatial extent of the significantly activating brain regions (i.e., narrow area of activation vs. widespread activation across brain regions).

This paper is divided into three analyses:

1. First, we study the characteristics of the hemodynamic response time courses during the social stimuli at each session from 5 months to 5 years of age.
2. Second, we study the spatial distribution of brain activation during the social task cross-sectionally at each session from 5 months to 5 years at the group-level.
3. Third, we extract participants’ longitudinal trajectories from 5 months to 5 years, creating individual profiles to explore the neural bases underlying the differences between participants’ social specialization.

## 2 Methods

### 2.1 Participants

Participants for this study were recruited as part of the BRIGHT Project (Lloyd-Fox et al., 2024), from villages across the rural West Kiang District in The Gambia, where a field station of the MRC Unit The Gambia at the London School of Hygiene and Tropical Medicine is based. Ethnicity in the West Kiang District of The Gambia is predominantly Mandinka (79.9 % of the population (Hennig et al., 2017)) with its unique language and cultural characteristics. The local population commonly experience seasonal food insecurity, and infections and other health challenges are common, especially among infants and young children, often leading to growth faltering (Nabwera et al., 2017; Schoenbuchner et al., 2019).

Demographics from the full Gambian cohort in the BRIGHT Project can be found in Lloyd-Fox et al. (2024): in summary, families live in multigenerational households with up to 36 members per compound. Polygamy was common within the cohort with 38.8 % of fathers having more than one wife. Consequently, while mothers had on average 4.4 children, including the infant enrolled in the study, fathers had on average 6.9, with a range of 1–23 children attributed to a single father. For the generation of parents within our cohort, formal schooling was not readily available when they themselves were children; therefore, on average, mothers and fathers within the study had completed 3 and 4 years of schooling respectively. To avoid confounds caused by multiple translations of the study protocols, only families where Mandinka was spoken as the first language were recruited into the study. In addition, 16.7 % of primary caregivers reported that they spoke a second language and 3.5 % of primary caregivers spoke three languages.

Participating families were recruited into BRIGHT antenatally, were seen at home for a neonatal examination in the immediate postpartum period, and were then invited to attend scheduled assessments at 7 timepoints post-partum (7–14 days (at home), 1, 5, 8, 12, 18 and 24 months of infant age). The same cohort was then followed up at a single further timepoint when they were aged between 3 and 5 years of age (the BRIGHT Kids study). 204 infants were initially enrolled at 1 month of age.

Ethics approval was obtained from the joint Gambia Government - MRC Unit The Gambia Ethics Committee (SCC 1351 for BRIGHT and Project reference 22737 for BRIGHT Kids), and informed consent was obtained from carers/parents in writing or via thumbprint if they were unable to write.

### 2.2 Experimental design

The auditory-visual social perception paradigm used for this study was first described by Lloyd-Fox, Papademetriou, et al. (2014). This was the first paradigm presented to participants as part of a battery of fNIRS tasks (Lloyd-Fox et al., 2024) implemented at the 5, 8, 12, 18, 24 months, and 3–5 years study visits. fNIRS data for the social perception task were collected from 140 infants at 5 months of age; 126 at 8 months; 132 at 12 months; 121 at 18 months; 130 at 24 months; and 163 at 3–5 years. The task was also presented at 1 month, but that session was not included here as the paradigm was different, with infants being asleep and no *visual social (silent)* condition.

**Fig. 1.**
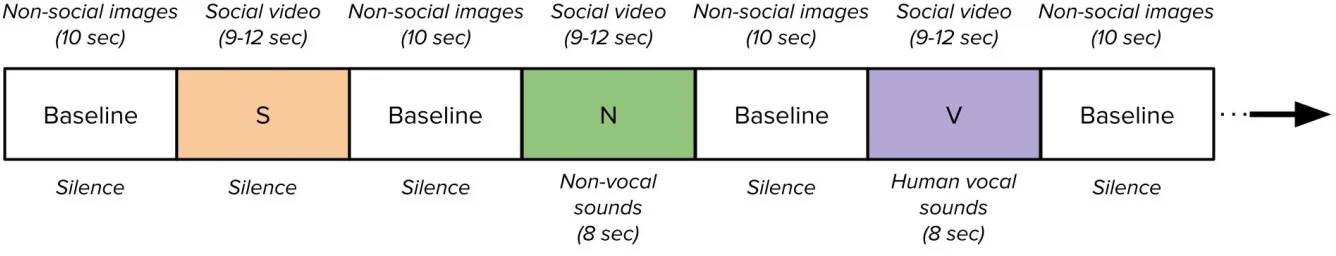
Illustration of the social task experiment paradigm showing the sequence of trials with duration of the stimuli (S: visual social (silent); N: auditory non-social (non-vocal); V: auditory social (vocal)).

The trials were presented with a period of baseline between each. The sequence of stimulus presentation has been used in previous research (Blanco et al., 2023; Lloyd-Fox et al., 2012, 2013) and is presented in Figure 1.

Three conditions (*visual social (silent)*, *auditory social (vocal)*, *auditory non-social (non-vocal)*) were presented in the same pseudo-randomized order across infants in a repeating loop (*visual social (silent)* → *auditory non-social (non-vocal)* → *auditory social (vocal)* → *visual social (silent)* → *auditory social (vocal)* → *auditory non-social (non-vocal)* →…) of trials until the infants became bored or fussy as judged by the experimenter who was monitoring their behaviour, up to a maximum of 8 trials per condition. During each experimental trial, a silent social video of 9 to 12 seconds with a local actor performing nursery rhyme gestures was played. These consisted of full-colour, life-size (head and shoulders only) social videos of adults (Gambian nationals) who either moved their eyes left or right or performed hand games: “Peek-a-boo” and “Incy-Wincy Spider”. To ensure infants’ continuous attention, especially since the social visual stimuli was also presented during auditory trials, there were six different visual social videos (two actors; three types of social video). These visual stimuli were displayed on a 24-inch plasma screen with a viewing distance of approximately 1 meter. For the *auditory social (vocal)* and *auditory non-social (non-vocal)* trials, auditory cues were also played at the onset of the trials with a duration of 8 seconds. The content and duration of the social videos were not synchronised with the auditory stimuli. The 8-second auditory stimuli consisted of four different sounds (of vocal or non-vocal stimuli) presented for 0.37–2.92 seconds each, interleaved by short silence periods (of 0.16–0.24 seconds). The two auditory conditions were equivalent in terms of average sound intensity and duration (p-value = 0.65). In the *auditory social (vocal)* condition, the video was accompanied by non-speech vocalizations (sequence of coughing, crying, laughing and yawning). In the *auditory non-social (non-vocal)* condition, it was environmental sounds (that were not human or animal produced) familiar to the participants (sequence of running water, bells, and rattles). In the *visual social (silent)* condition, the video was not accompanied by any sounds. A restriction of studying auditory processing in awake infants of this age is that they need to be presented with concurrent visual stimulation to reduce infant movement and thus artefact in the signal. Therefore, in line with previous work using these stimuli, we chose to employ the same visual stimuli during the presentation of the auditory stimuli as the one used when auditory stimulation was absent. Each experimental condition was separated by a baseline of 10 seconds with a pseudo-random sequence of images of objects, amenities or environment familiar to the participants, each image presented for 1–3 seconds.

Videos of the participants were recorded during the task, and participants’ gaze was monitored using an eye tracker (Tobii TX-300, Tobii AB, Sweden). This enabled the identification of trials during which the participants did not pay attention to the task. Trials with less than 60 % engagement were discarded from the analysis.

### 2.3 Data acquisition

**Fig. 2.**
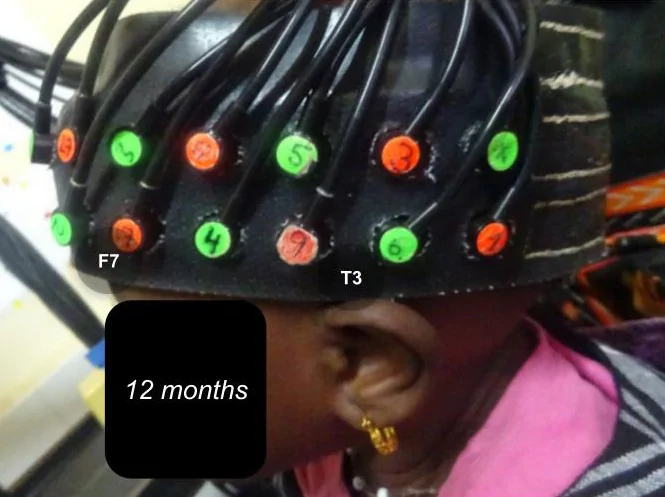
NTS fNIRS System used for the BRIGHT study with reference 10–20 positions for participants aged between 5 months to 3–5 years (12 months example on the photo).

fNIRS data were collected using a continuous wave NTS fNIRS System (Gowerlabs Ltd. London, UK) with two wavelengths of 780 and 850 nm, and a sampling frequency of 10 Hz (Everdell et al., 2005). The fNIRS sensor included 12 sources and 14 detectors for a total of 34 channels located over the frontal and temporal brain regions bilaterally for participants aged between 5 months and 3–5 years (Fig. 2). At 3–5 years, new optical fibres were used, and the custom-made silicone band holding the optode arrays was replaced by a modified EasyCap (EASYCAP GmbH), which facilitated replicability of headgear positioning with the older participants who had more hair than young infants.

Before starting the session, infants’ head measurements were taken (head circumference, ear-to-ear measurements over the forehead and over the top of the head) to guide alignment of the headgear with the 10–20 system anatomical landmarks (pre-auricular points and nasion) (Lloyd-Fox, Richards, et al., 2014). Once the headgear was securely placed on the participant’s head, photographs of the infants wearing the headgear were taken. Whenever possible, photographs were taken at the end as well. Pictures were used to assess the headgear placement, enabling us to reject recordings for which the displacement of reference optodes was greater than 1.6 cm based on the known inter-optode distance (Blasi et al., 2014). The array was registered onto age-appropriate brain templates with anatomical labels (Collins-Jones et al., 2021). This co-registration was completed for all time points except the 3–5 years age group. However, as after a phase of rapid growth of the brain in infancy (reaching 80 % of the adult size at 24 months) the rate of change of brain structure slows (Gilmore et al., 2018), we assumed that channel mapping would not change greatly between 24 months and 3–5 years. Consistent with this, head circumference at 24 months (46.58 ± 1.47 cm) measured about 95 % of the head circumference at 3–5 years (49.20 ± 1.43 cm) (see Table 1). The cortical areas that the channels covered for infants aged 5 months to 3–5 years ranged from the inferior frontal gyrus at the front of the array, to the posterior sections of the superior, middle, and inferior temporal gyri at the back of the array.

**Table 1.** Participant information describing for each session the number of participants with data analysed (N), the percentage of male and female participants, the mean age ± standard deviation (SD) at the session calculated from the date of birth (in months), the mean ± SD weight-for-height Z-score (WHZ), the mean ± SD head circumference (HC, in cm), the mean ± SD number of retained channels (out of a total of 34), and the mean ± SD number of retained trials per condition (out of a total of 8 maximum).

Session	N	Male/female	Age (months)	WHZ	HC (cm)	Number of channels	Trials per condition
5 mo	127	48%/52%	5.26 ± 0.30	-0.19 ± 1.01	41.28 ± 1.25	30.61 ± 3.43	5.55 ± 0.92
8 mo	110	50%/50%	8.11 ± 0.37	-0.37 ± 1.03	42.85 ± 1.34	30.79 ± 3.06	5.14 ± 0.83
12 mo	116	49%/51%	12.23 ± 0.45	-0.57 ± 1.02	44.39 ± 1.37	30.56 ± 2.81	5.20 ± 0.93
18 mo	111	52%/48%	18.34 ± 0.60	-0.60 ± 0.93	45.91 ± 1.48	29.70 ± 3.46	5.14 ± 0.86
24 mo	112	46%/54%	24.39 ± 0.77	-0.42 ± 0.94	46.58 ± 1.47	28.71 ± 3.68	5.38 ± 0.66
3–5 years	124	56%/44%	48.98 ± 6.92	-0.78 ± 0.93	49.20 ± 1.43	32.26 ± 2.86	5.67 ± 0.44

### 2.4 fNIRS data pre-processing and quality control

The data processing followed the pipeline described by Bulgarelli and colleagues (Bulgarelli et al., 2020) and was implemented in MATLAB using Homer2 (Huppert et al., 2009). First, channels with very low intensity (lower than $3.10^{-4}$ , specific to the NTS fNIRS system) or low signal quality assessed using QT-NIRS (SCI = 0.7, cardiac power = 0.1 µV) (Hernandez & Pollonini, 2020; Pollonini et al., 2014) were discarded. Light intensities were converted into optical densities, and then motion artefacts were detected using Homer2 to be corrected using a combination of Spline interpolation (p = 0.99) and wavelet filtering (iqr = 0.8) as previously suggested (Di Lorenzo et al., 2019). Trials that still showed evidence of motion artefacts after correction were then rejected. Moreover, trials that were attended for less than 60 % by the infants were also rejected. Data were bandpass filtered (0.02–0.6 Hz) in order to remove instrumental and systemic noise while keeping frequencies related to the hemodynamic response function (HRF) and experiment paradigm (around 0.1 Hz). We then converted the signal into HbO and HbR concentration changes using the modified Beer-Lambert law (Delpy et al., 1988) and baseline corrected using the pre-trial baseline of 2 seconds prior to the trial trigger onset. Lastly, concentration changes were block averaged up to 20 seconds post-stimulus onset and baseline corrected using 2 seconds prior to the stimulus onset. The block averaging method was preferred here compared to the GLM, given its simplicity and the evidence that both approaches eventually lead to similar conclusions (Luke et al., 2021). Participants were excluded from data analyses at a specific session if they had fewer than 3 remaining trials per condition or less than 60 % valid channels after preprocessing and looking time analyses.

### 2.5 First analysis: Profiling the fNIRS responses

Statistical analyses were performed using a combination of in-house Python 3 scripts based on *SciPy* (Virtanen et al., 2020) and *statsmodels* (Seabold & Perktold, 2010).

Firstly, we examined the characteristics of the haemodynamic response to the stimuli by block averaging each infant’s hemodynamic responses to the different conditions (*visual social (silent)*, *auditory non-social (non-vocal)*, *auditory social (vocal)*) within each session. We explored whether we would observe age-related and stimulus-related differences in haemodynamic profiles. To investigate whether the magnitude of the hemodynamic response to the presentation of the experimental condition trials (relative to the previous baseline condition response) was influenced by the age point and stimulus type, we extracted the average maximum amplitudes for HbO and average minimum amplitudes for HbR for each infant and ran two-way ANOVA tests. To investigate the latency of the peak response, we examined the time at which the peak (maximum HbO or minimum HbR) of the hemodynamic response profile was observed for each stimulus type and age point and tested significance again with two-way ANOVA tests.

### 2.6 Second analysis: Cross-sectional group level analysis

Following up the profiling of fNIRS HRFs, we investigated brain responses to social stimuli cross-sectionally at the group level. This was done using the mean HbO and HbR concentration changes over a 4-second range around the average time-to-peak (i.e. HbO time-to-peak was determined by averaging all trials and channels within a given condition at the group level, also averaged across the two compared conditions in the case of a contrast). A 4-second span was selected to include a sufficient individual variability of time-to-peak around the average time-to-peak across participants. The choice of extracting the mean around the expected peak and not on a fixed time window was motivated by limitations from previous work. Indeed, in our previous feasibility research using the same visual and auditory stimuli within this rural Gambian population we found preliminary evidence of differences in the timing and shape of the hemodynamic response, which potentially also differed between social and non-social conditions and age of participant (Lloyd-Fox et al., 2017). Therefore, our approach decreased the likelihood of preferentially biasing one condition over another, or one age point over another.

One-way channel-by-channel comparisons were performed using t-tests to assess brain activation during the *visual social (silent)* condition (one-sample t-tests, to investigate the response to *visual social (silent)* relative to the final 2 seconds of the previous non-social baseline trial) or the contrast between *auditory social (vocal)* and *auditory non-social (non-vocal)* conditions (paired t-tests). Activation was defined as a statistically significant increase in HbO and/or statistically significant decrease in HbR with a p-value smaller than 0.05. False discovery rate (FDR) correction (Benjamini & Hochberg, 1995) was performed at the channel level (34 tests, one per channel), for each chromophore separately. For the auditory contrast more specifically, we defined a selectivity towards *auditory social (vocal)* as a significant activation for *auditory social (vocal)* coupled with a greater response to *auditory social (vocal)* than *auditory non-social (non-vocal)*.

### 2.7 Third analysis: Longitudinal analysis of individual cortical specialisation

#### 2.7.1 Feature extraction

**Appendix Fig. A1.**
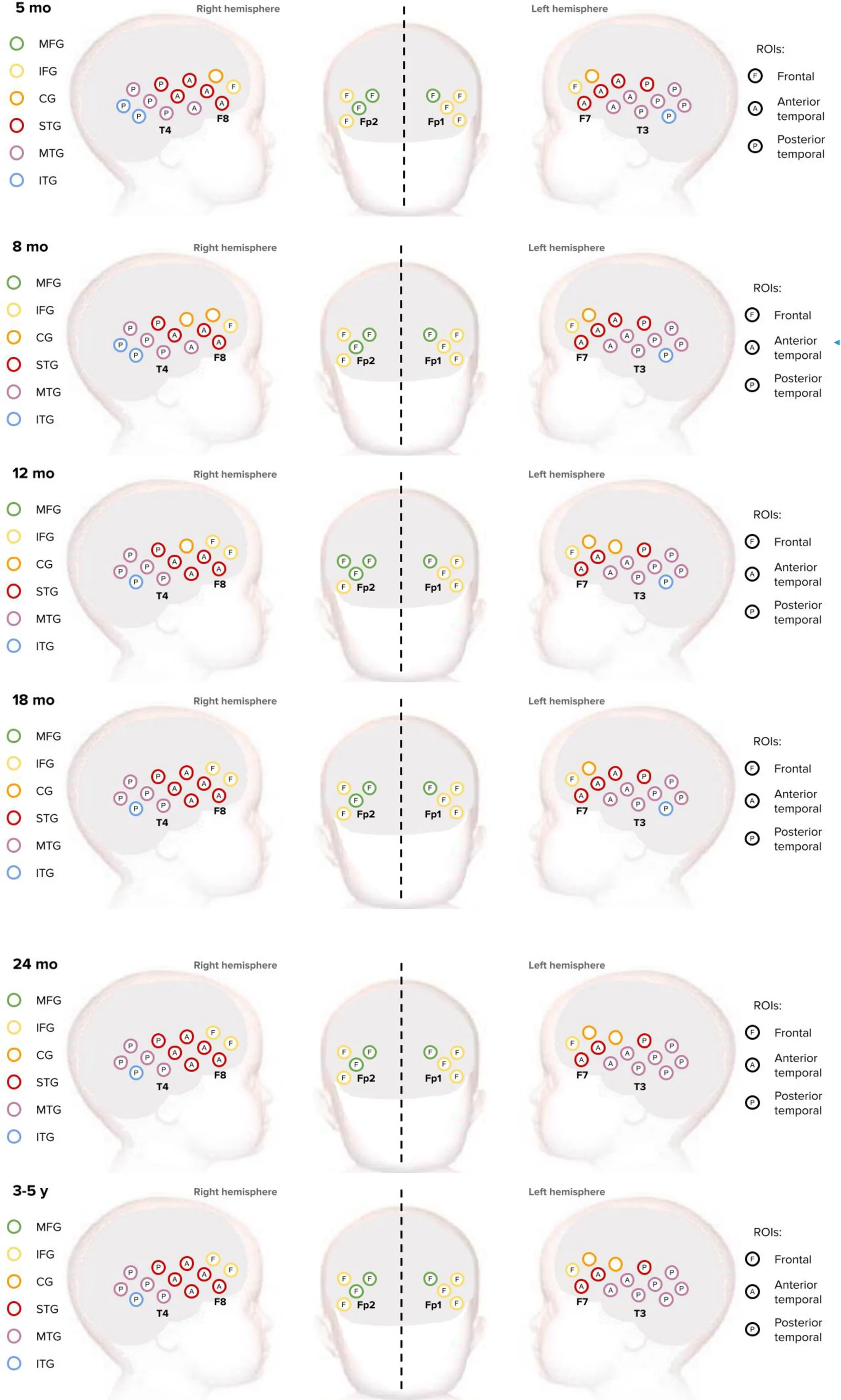
Regions of interests used for the longitudinal analysis (frontal, anterior temporal, posterior temporal) along with the anatomical co-registration of optodes: middle frontal gyrus (MFG), inferior frontal gyrus (IFG), pre/postcentral gyrus (CG), superior temporal gyrus (STG), middle temporal gyrus (MTG), inferior temporal gyrus (ITG).

 Following the cross-sectional analysis, we aimed to investigate age-related changes in auditory social selectivity, with the comparison of the *auditory social (vocal)* and *auditory non-social (non-vocal)* stimuli at the participant level. Here, we focused on HbO as a biomarker of social selectivity: this was driven by the fact that it has a higher contrast-to-noise ratio than HbR (Strangman et al., 2002), especially with infants (Cristia et al., 2013), which therefore makes it a more suited feature to use at the individual level. This choice was further backed by our analysis of the fNIRS responses which confirmed that task responses were positive in HbO and negative in HbR (Fig. 6), without signs of major motion artefacts. Based on co-registration (Collins-Jones et al., 2021) for each hemisphere, the optode array was divided into three regions of interest from anterior to posterior (see Appendix Fig. A1): the *frontal* region of interest was composed of the middle/inferior frontal gyri; the *anterior temporal* region of interest was composed of the anterior temporal gyri (up to the T4/T3 10-20 landmark); the *posterior temporal* region of interest was composed of the posterior temporal gyri (from the T4/T3 10-20 landmark and back). This enabled us to average channels by region of interest in order to study comparable anatomical brain regions across ages, accounting for head growth and head shape changes. Each region of interest ranged from 4 to 6 channels, specific enough to investigate previously identified regions of interest from literature (Pernet et al., 2015), but with enough channels to mitigate missing channels and the use of fMRI templates as opposed to fMRI scans from each participant. Selectivity towards social stimuli was defined as an anterior temporal HbO response which was positive for the *auditory social (vocal)* condition and greater for the *auditory social (vocal)* than *auditory non-social (non-vocal)*. Selectivity toward non-social stimuli was defined as an anterior temporal HbO response which was positive for the *auditory non-social (non-vocal)* condition and greater for the *auditory non-social (non-vocal)* than *auditory social (vocal)*. The remaining participants were defined as non-selective towards auditory social (vocal) nor auditory non-social (non-vocal).

#### 2.7.2 Individual social selectivity responses across ages

Using the extracted categorical features, we described the evolution of the proportion of participants showing a social selectivity across ages, and used a Bayesian Generalized Linear Mixed Model (GLMM) with a binomial link function to analyse the relationship between age and the social selectivity binary outcome (auditory social selectivity or not). The model included random intercepts for participants to account for within-subject variability. Then, to describe the social selectivity responses further, we investigated the evolution of social selectivity responses at the individual level for participants with valid data at each session during the first year of life (5, 8 and 12 months, N = 50) in each of the 3 regions of interest bilaterally.

#### 2.7.3 Developmental trajectories

Finally, continuing analysis with the 50 participants with full data over the first year of life, we proposed a way to group participant into social selectivity profiles based on their age of first social selectivity: either first social selectivity at 5 months, 8 months, 12 months, or categorised as a *remaining group* if they did not show social selectivity by then. This enabled us to visualise their brain response trajectories to explain the different social selectivity profiles.

## 3 Results

**Fig. 3.**
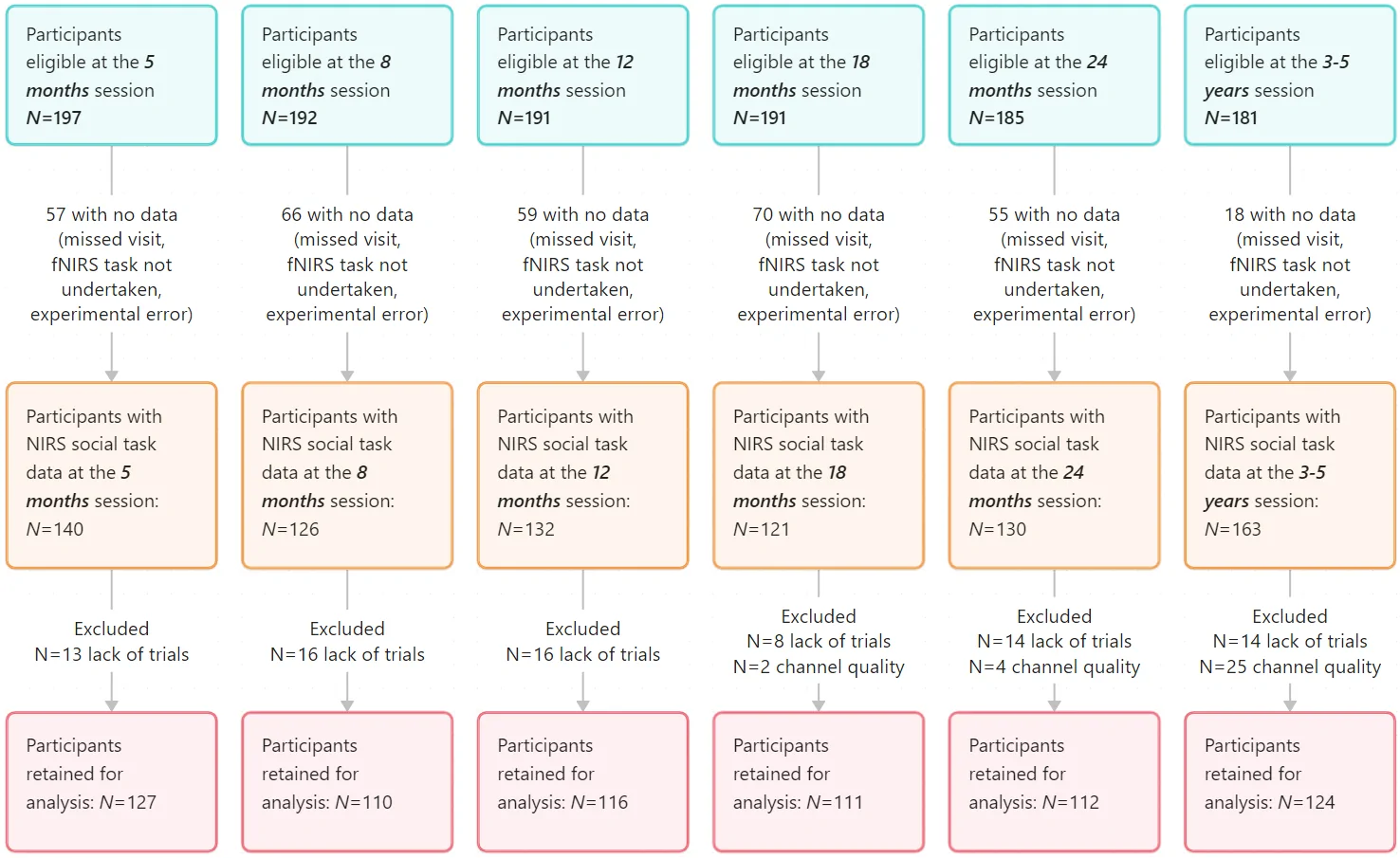
Flowchart of available fNIRS data at each age point.

Overall, the number of participants with valid fNIRS data varied from 110 to 127, depending on the session. The detail of sample retention and attrition is presented in a flowchart in Figure 3. Of those infants who took part in the study at each age point data quality was overall high with retention ranging from 76 to 92%, showing that these paradigms are suitable for use across this age range. Participant characteristics for each session are presented in Table 1.

**Fig. 4.**
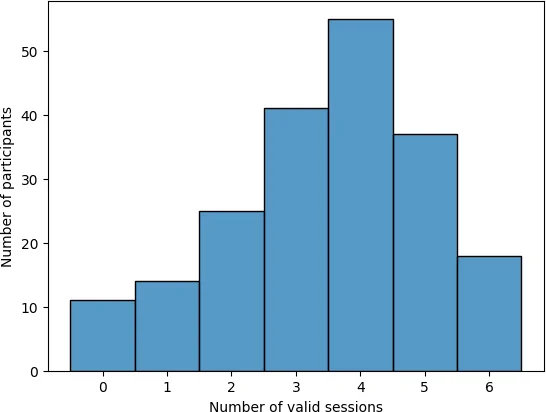
Histogram showing how many participants contributed valid data across the six NIRS sessions at 5, 8, 12, 18, 24 months and 3–5 years.

On average, participants had 3.5 sessions with fNIRS data retained for analysis, with 110 participants having valid fNIRS data on 4 sessions or more between 5 months and 3–5 years (Fig. 4).

### 3.1 First analysis: Profiling the fNIRS responses

**Fig. 5.**
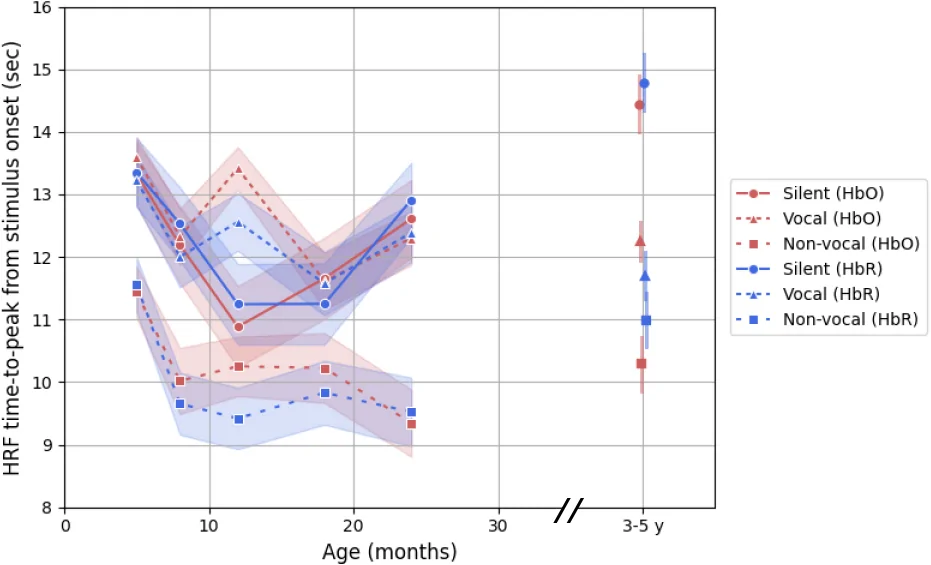
Time-to-peak from stimulus onset for the visual social (silent), auditory social (vocal) and auditory non-social (non-vocal) conditions (HbO and HbR) across ages (brands represent the standard error across participants).

To study the hemodynamic response function (HRF) peaks we first investigated the time-to-peak for the different conditions at different ages (Fig. 5).

The two-way ANOVA (for the condition and age point) for HbO and HbR showed significant effects of both the conditions and the age points (all p-values < 0.05). These results led us to use, for the study of activation, an approach centring the HRF window averaging around the mean time to peak (as opposed to a constant set time window). Therefore, a 4-second time window centred around the average HbO time-to-peak across channels and cohort for each condition and at each age point was selected to compute the average haemoglobin concentration changes for each trial for further statistical analysis. For the analysis of the contrast between *auditory social (vocal)* and *auditory non-social (non-vocal)*, the average time-to-peak across the two conditions was used to centre the time window used to extract the mean HRF concentration changes, which ranged from 8.8*–*12.8 seconds post-stimulus onset to 10.5*–*14.5 seconds depending on the age points (Table 2). For the analysis of the *visual social (silent)* condition, the average time-to-peak on that condition was used, which resulted in a time windows ranging from 8.9*–*12.9 seconds post-stimulus onset for the earliest to 12.4*–*16.4 seconds for the latest (Table 2).

**Table 2.** Time window around the average HbO time-to-peak used across different age points in seconds post-stimulus onset for the auditory social (vocal) > auditory non-social (non-vocal) contrast and visual social (silent) condition.

Session	Auditory vocal > non-vocal	Visual social (silent)
5 mo	10.5–14.5 seconds	11.3–15.3 seconds
8 mo	9.2–13.2 seconds	10.2–14.2 seconds
12 mo	9.8–13.8 seconds	8.9–12.9 seconds
18 mo	8.9–12.9 seconds	9.7–13.7 seconds
24 mo	8.8–12.8 seconds	10.6–14.6 seconds
3–5 years	9.3–13.3 seconds	12.4–16.4 seconds

**Fig. 6.**
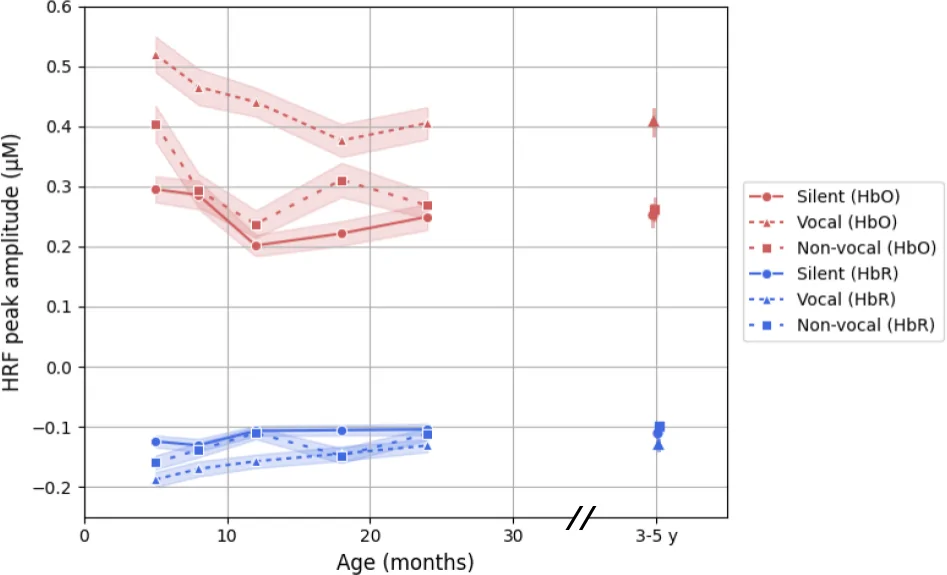
Value of the peak (in µM) for the HRF of the visual social (silent) auditory social (vocal) and auditory non-social (non-vocal) conditions (HbO and HbR) across ages (in months) compared to the pre-trial baseline of 2 seconds (bands represent the standard error across participants).

We also studied the absolute peak amplitudes of the HbO and HbR HRFs across age points for which a graphical representation can be seen in Figure 6. The two-way ANOVA tests for HbO and HbR did show a significant effect of both the conditions and the age points on the absolute maximum magnitude of the HRFs (all p-values smaller than 0.01).

### 3.2 Second analysis: Cross-sectional group level analysis

#### 3.2.1 Visual social (silent)

**Fig. 7.**
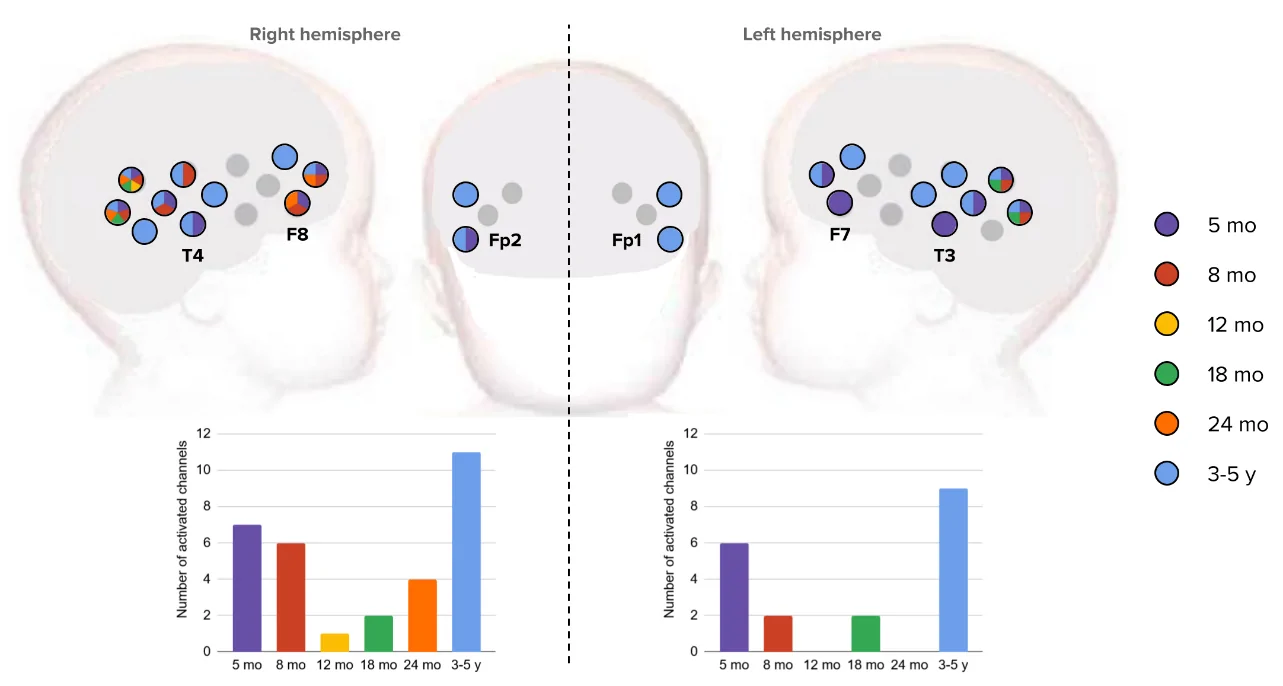
Graphical representation of the channels that showed significant activation to the visual social (silent) condition (significant increase in HbO and/or significant decrease in HbR). Each channel location shows the age points for which significant activation was detected (see legend for colour coded age-related responses). The bar charts represent the evolution of the number of channels with this significant contrast activation for each hemisphere across ages.

With group-level analysis at each of the age points (channel by channel t-tests, FDR corrected), we found that the visual social stimuli elicited activation in channels covering regions known to be involved in social perception across all age points. The topographic map of brain activation can be seen in Figure 7. Overall, the number of significant channels decreased during infancy between 5 and 24 months, suggesting the response becomes more localised over the posterior middle/superior temporal gyri and inferior frontal gyri. 13 channels showed statistically significant activations at 5 months, 8 channels at 8 months, and 1–4 between 12–24 months. Interestingly, the number of channels activated at 3–5 years increased to 20 channels. Results showed a selective response for the *visual social (silent)* stimuli over the right posterior temporal region across all six time points from 5 months to 3–5 years of age (with 4 of 6 ages also showing activation in this region of the left hemisphere). Inferior frontal regions showed age-related changes in brain activation with the youngest (≤ 8 months) and oldest (24 months to 3–5 years) time points evidencing selective social visual responses. In fact, just under 50 % of the active channels at 3–5 years were found in inferior frontal regions, which drove the pattern of developmental change to an increase in the overall number of channels active at this age.

#### 3.2.2 Auditory social (vocal) versus non-social (non-vocal)

For the contrast between *auditory social (vocal)* and *auditory non-social (non-vocal)*, stimuli (using paired t-tests, FDR corrected), we observed greater activation in response to vocal compared to non-vocal stimuli across all ages.

Contrary to the social visual selective responses, auditory selective responses revealed a stronger and more prolonged response to vocal compared to non-vocal stimuli across all six age points from 5 months to 3–5 years old.

Initially, at 5 months, 8 channels showed a greater response to *auditory social (vocal)* than *auditory non-social (non-vocal)*, increasing to 10 at 8 months and 27 at 12 months. The number of channels with a significant contrast then decreased to 3 at 18 months and increased again to 23 and 21 channels at 24 months and 3–5 years respectively.

**Fig. 8.**
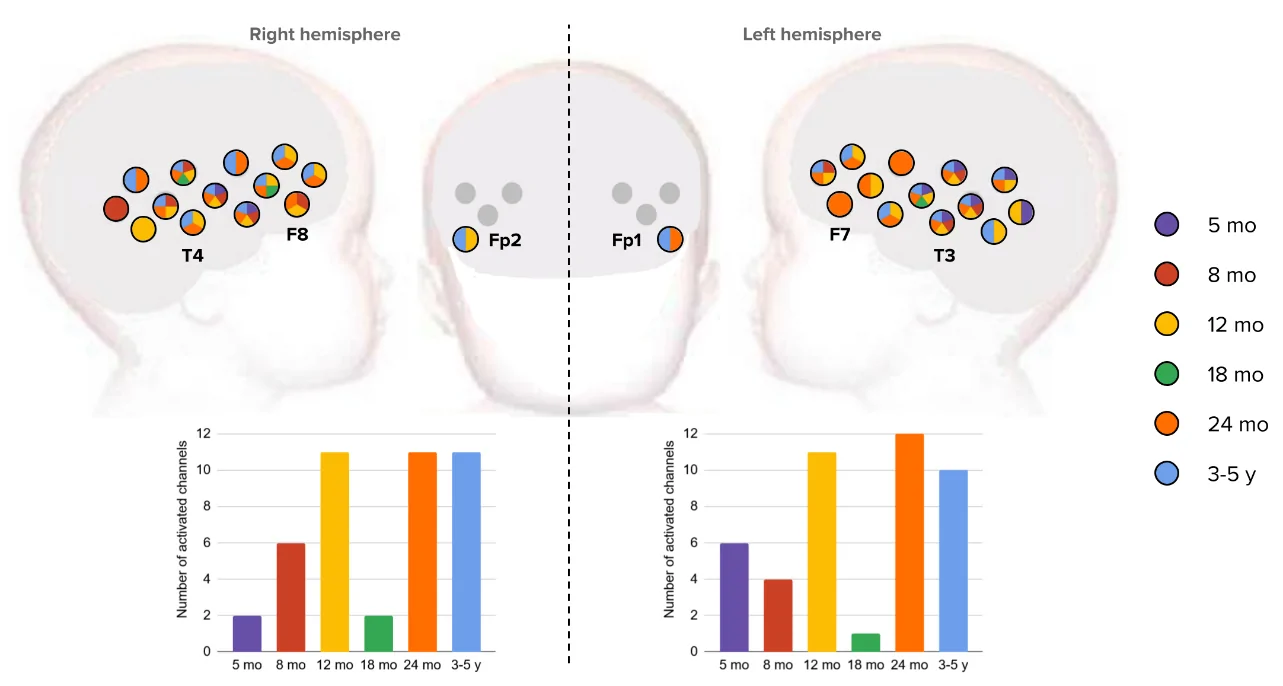
Graphical representation of the channels that showed significant activation on the auditory social (vocal) > auditory non-social (non-vocal) contrast (significant increase in HbO and/or significant decrease in HbR). Each channel location shows the age points for which significant activation was detected (see legend for colour coded age-related responses). A mask was applied such that we considered a significant activation on the contrast only if there was a significant activation to auditory social (vocal) compared to baseline in the first place. The bar charts represent the evolution of the number of channels with this significant contrast activation for each hemisphere across ages.

**Fig. 9.**
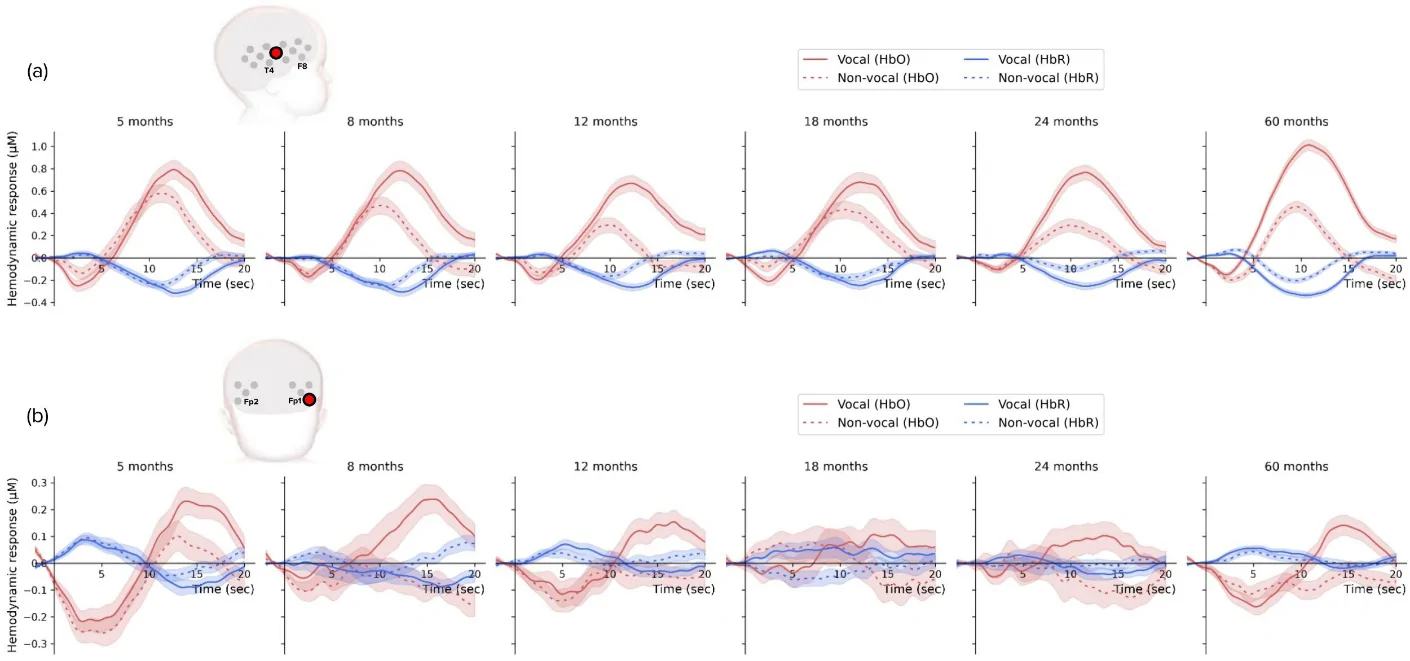
Examples of hemodynamic responses for the auditory social (vocal) and auditory non-social (non-vocal) conditions across ages, (a) in a right superior temporal gyrus channel which revealed social selectivity at all ages except 18 months, and (b) in a left inferior frontal gyrus channel which revealed social selectivity at 24 months and 3–5 years. Coloured shade bands represent the standard error.

**Appendix Fig. A2.**
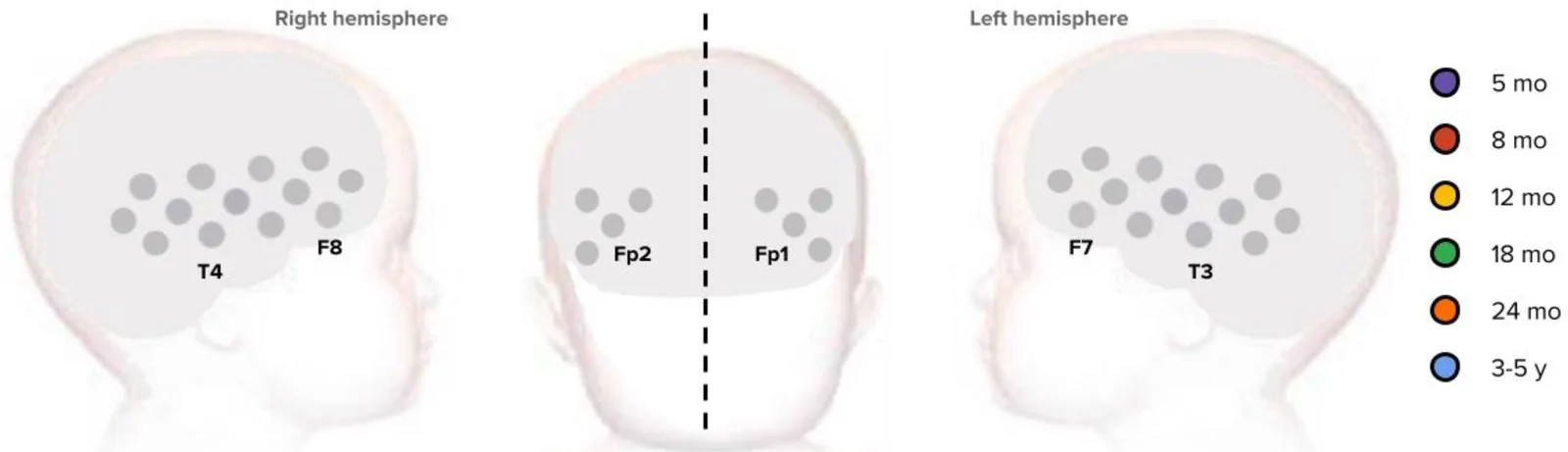
Graphical representation of the channels that showed significant activation on the auditory non-social (non-vocal) > auditory social (vocal) contrast (significant increase in HbO and/or significant decrease in HbR). A mask was applied such that we considered a significant activation on the contrast only if there was a significant activation to auditory non-social (non-vocal) compared to baseline in the first place. No channel showed a significant contrast.

The selective response was observed in the temporal regions, especially around the superior temporal gyrus bilaterally, with an area of selective activation growing up at 8 months and 12 months, where we also observed a response in the inferior frontal gyri bilaterally. The selective response became much more localised at 18 months, and became again more widespread around the superior temporal gyrus at 3–5 years. The topographic representation of activation can be seen in Figure 8. The bilateral anterior temporal regions show consistent activation across all six time points, localized in the anterior superior and middle temporal gyri. We also studied the inverted contrast which showed *no auditory non-social (non-vocal)* response greater than *auditory social (vocal)* for any of the channels at any of the ages from 5 months to 3–5 years (the graphical representation can be seen in Appendix Fig. A2). Examples of hemodynamic responses across ages can be found in Figure 9, with a channel in the right superior temporal gyrus that showed a significantly greater response to *auditory social (vocal)* than *auditory non-social (non-vocal)* at all sessions except 18 months (Fig. 9a). Note however that two neighbouring channels in this hemisphere did show social selectivity at 18 months, and the homologous channel in the left hemisphere also revealed significant selectivity at that age. We also show in Figure 9b the hemodynamic responses of a left inferior frontal gyrus channel with a significant contrast only at 24 months and 3–5 years. While some portions of the time courses may look different across conditions, significant differences on the contrasts were considered only on the 4-second time windows specific to each age (see Table 2) to avoid bias towards any of the conditions.

### 3.3 Third analysis: Longitudinal analysis of individual cortical specialisation

#### 3.3.1 Individual social selectivity responses across ages

**Fig. 10.**
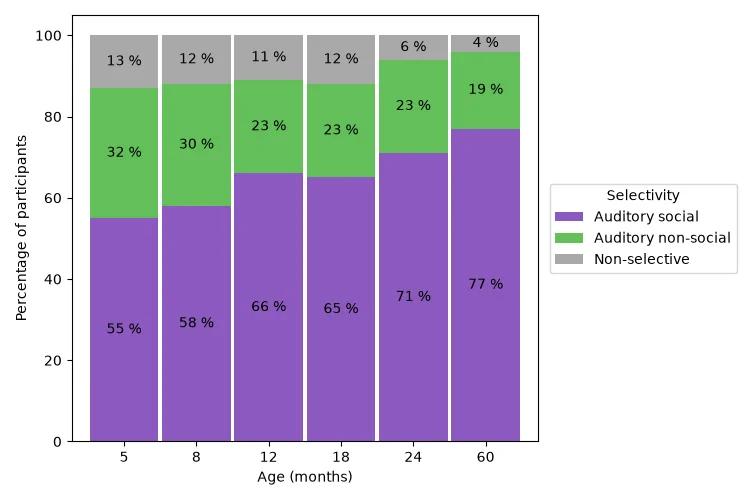
Bar plots showing the number of participants who showed selectivity towards each condition at each age point (auditory social (vocal) selectivity, auditory non-social (non-vocal) selectivity, or no selectivity). Selectivity toward a condition is defined as an HbO activation in response to that condition greater than the other condition in the anterior temporal regions of interest. Participants who did not have HbO activation in response to any of the conditions were represented as non-selective. Number of participants: 127 at 5 months, 110 at 8 months, 116 at 12 months, 111 at 18 months, 112 at 24 months, 124 at 3–5 years.

We visualised the proportion of participants showing social or non-social selectivity at each age point (Fig. 10). This demonstrates that a greater proportion of participants showed selectivity towards social stimuli as they grew older: from 55% of the participants showing social selectivity at 5 months to 77% at 3–5 years. When having a closer look at the data to try to understand how participants moved between the different selectivity groups across ages, out of the 41 infants with an *auditory non-social (vocal)* selectivity at 5 months, we saw that most of them (65.9 %) showed vocal selectivity in the majority of sessions after (from 8 months to 3–5 years). We also observed that only 2 participants showed only non-social selectivity or no selectivity at every age point from 5 months to 3–5 years.

In studying the relationship of social selectivity with age with the Bayesian GLMM (Table 3), we found that the posterior mean for the fixed effect of age was 0.0246 with a posterior standard deviation of 0.0037 which suggests that an increase in age is associated with a higher probability of a social selectivity. The 95% credible interval for age (fixed effect) did not include zero, indicating that this effect is statistically significant. Additionally, the intercept term had a posterior mean of 0.2312 with a posterior standard deviation of 0.0831 which suggests that, at even before 5 months the log-odds of a social selectivity are slightly positive. Finally, the random intercepts for participants (random effect) showed a posterior mean of -0.4700 with a posterior standard deviation of 0.0513. The standard deviation of the random effects was estimated to be 0.625, with a 95% credible interval ranging from 0.564 to 0.693. This indicates substantial variability in the intercepts across different participants.

**Table 3.** Posterior estimates for the Bayesian mixed generalised linear model with a binomial link function, showing the relationship between age and social selectivity with participants as random effect.

Parameter type	Posterior mean	Posterior standard deviation	Standard deviation	Standard deviation (lower bound)	Standard deviation (upper bound)
Intercept	0.2312	0.0831			
Age (months)	0.0246	0.0037			
Participant intercept	-0.4700	0.0513	0.625	0.564	0.693

The fixed effect of age quantifies the change in log-odds per unit increase in age, and the random intercepts account for variability across participants.

**Fig. 11.**
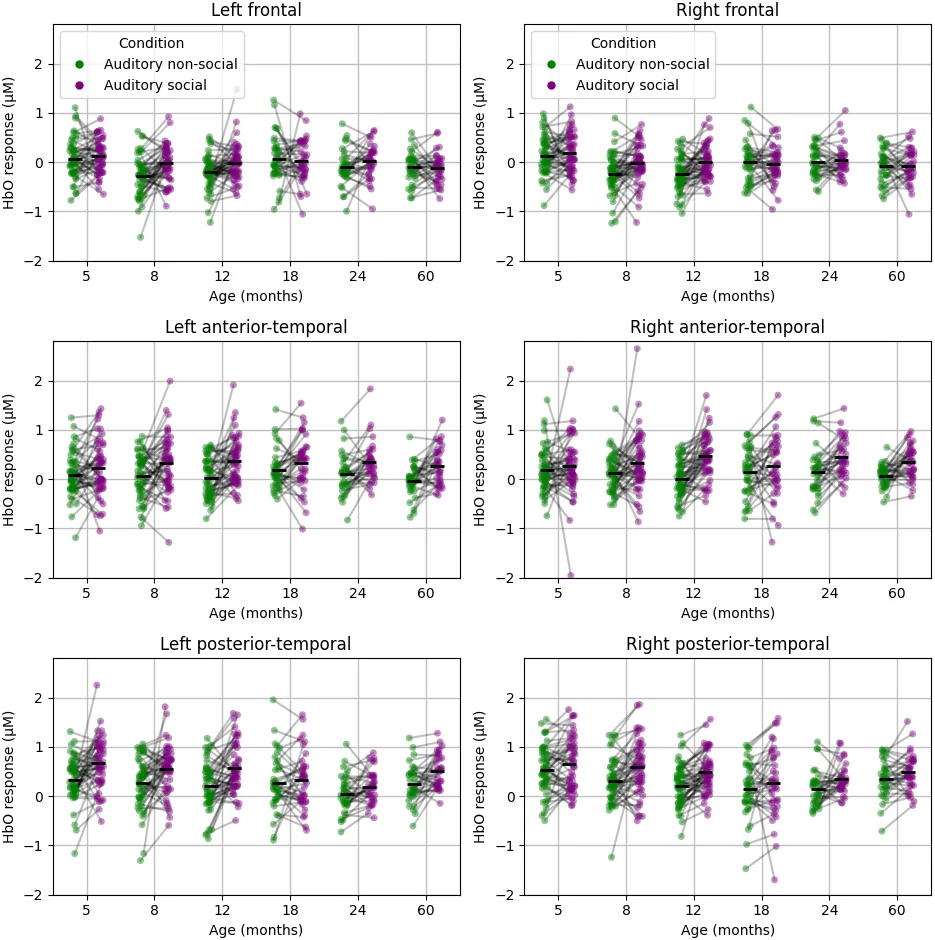
Graphical representation of the auditory social (vocal) and auditory non-social (non-vocal) HbO responses in the different regions of interest for each age point. Green dots represent individual auditory non-social (non-vocal) HbO responses while purple dots represent auditory social (vocal) HbO responses, with black marks representing the group averages. Links between the data points represent individuals.

We then investigated the 50 participants with data at all sessions during the first year of life with Figure 11 showing the HbO responses for the *auditory social (vocal)* and *auditory non-social (non-vocal)*, which enabled us to compare the response to the conditions for each participant specifically (link between data points).

The most consistent patterns of auditory social selectivity across participants were observed in anterior temporal regions (middle panels of Fig. 11), as most participants showed an increase between *auditory social (vocal)* and *auditory non-social (non-vocal)*. This was different than in the posterior temporal regions (bottom panels of Fig. 11) where the participants showed more varied trends.

More precisely, in the left anterior temporal region of interest, 60 % of participants showed a stronger response to *auditory social (vocal)* than *auditory non-social (non-vocal)* at 5 months (HbO average response of 0.23 µM for *auditory social (vocal)* and 0.09 for *auditory non-social (non-vocal)*), 66 % at 8 months (averages respectively 0.32 and 0.07 µM), 74 % at 12 months (0.37 and 0.02 µM), 65 % at 18 months (0.34 and 0.19 µM), 76 % at 24 months (0.36 and 0.10 µM), and 68 % at 3–5 years (0.28 and -0.03 µM).

In the right anterior temporal region of interest, 54 % of participants showed a stronger response to *auditory social (vocal)* than *auditory non-social (non-vocal)* at 5 month (HbO average response of 0.26 µM for *auditory social (vocal)* and 0.19 for *auditory non-social (non-vocal)*), 60 % at 8 months (respectively 0.34 and 0.13 µM), 84 % at 12 months (0.47 and 0.00 µM), 57 % at 18 months (0.27 and 0.14 µM), 76 % at 24 months (0.45 and 0.14 µM), and 71 % at 3–5 years (0.35 and 0.06 µM).

#### 3.3.2 Developmental trajectories

**Fig. 12.**
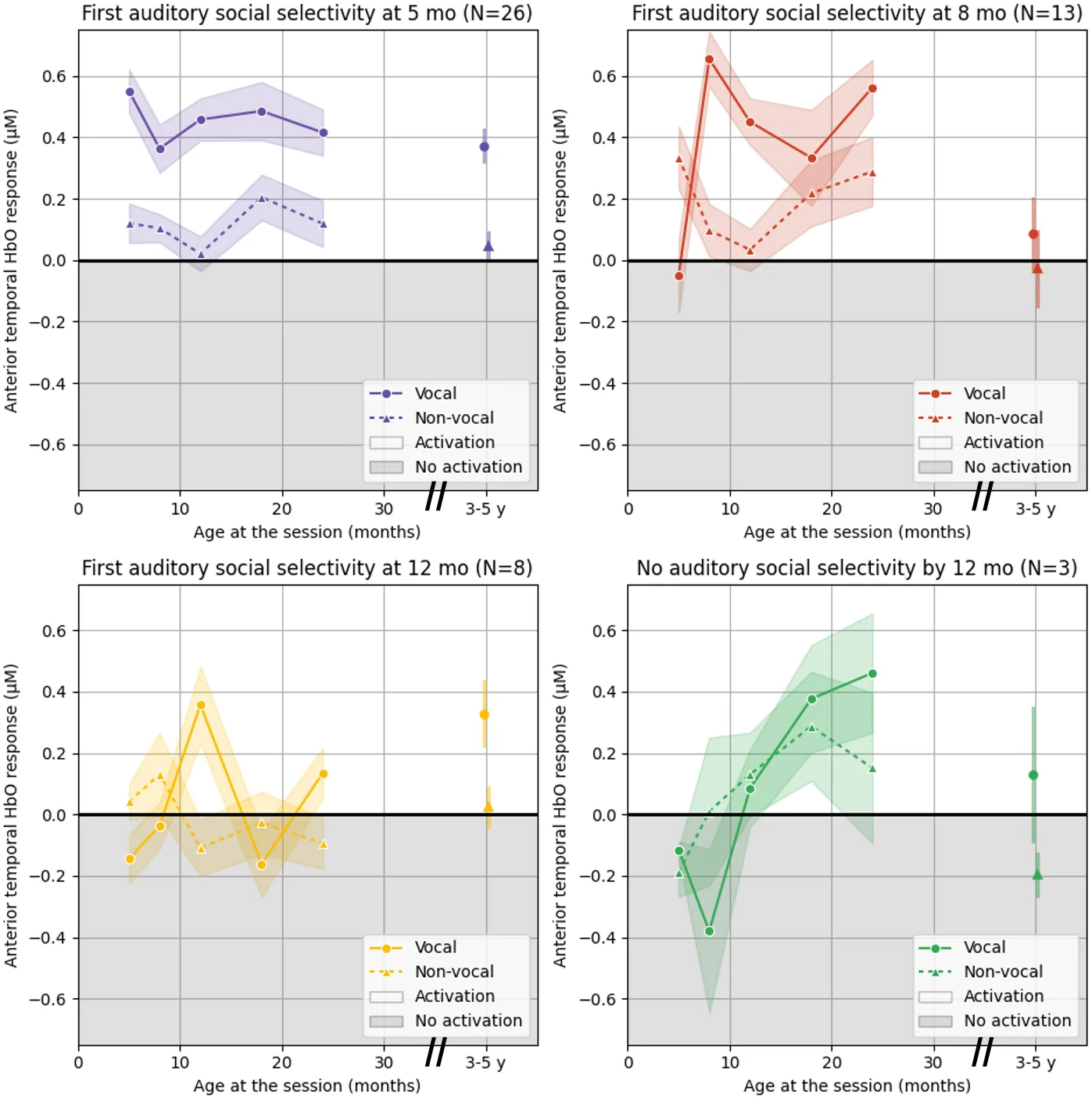
Graphical representations of the mean HbO responses (mean over a 4-second window centred around the cohort average peak response delays as described in Table 2) in anterior temporal regions for the auditory social (vocal) and auditory non-social (non-vocal) conditions across ages, for participants with data at all sessions between 5 and 12 months (N = 50). Each panel represents a separate social specialisation profile group based on the age of first auditory social selectivity. Coloured shade bands represent the standard error.

Finally, to further explore individual patterns of *auditory social (vocal)* > *auditory non-social (non-vocal)* we investigated the developmental trajectories of the 50 participants who had data at all sessions from 5 to 12 months (Fig. 12), with participants divided into groups based on the first age point of social selectivity (*auditory social (vocal)* > *auditory non-social (non-vocal)*) in the temporal anterior regions of interest. Of those 50 participants, 26 had their first social selectivity at 5 months (52.0 %), 13 at 8 months (26.0 %), 8 at 12 months (16.0 %), and 3 with no social selectivity by 12 months (6.0 %). The group of participants with first social selectivity at 5 months showed very clear responses to social stimuli greater than non-social stimuli at all the sessions. On the other hand, for participants with a first social selectivity at 8 and 12 months, we saw that even though they tended to remain overall socially selective after their first selectivity, the 18 months session seemed to be an exception, as seen in the previous group results with the group level showing significant contrasts in fewer channels (Fig. 8). Indeed, here there appeared to be a trend toward the inversion of the social selectivity at that age (non-social stimuli eliciting a greater anterior temporal brain response than social stimuli). Finally, the 3 participants out of the 50 with data at all sessions over the first year of life which did not show a social selectivity by age 12 months exhibited trajectories with much more variability.

## 4 Discussion

### 4.1 Social perception

This study, using data from a longitudinal study in rural Gambian infants, has shown that for visual social selectivity: (i) infants showed brain responses to visual social stimuli in the posterior temporal regions at 5, 8, 12, 18, 24 months and 3–5 years of age; (ii) a region of the right posterior temporal social brain network was consistently activated across all timepoints; (iii) additional inferior frontal brain regions were also activated but unlike temporal responses, different developmental patterns of activation were observed, with greater recruitment of this region when infants were younger (5*–*8 months), a drop in activation in mid-infancy, then a rise in late toddlerhood to pre-school age (2*–*5 years); and finally (iv) overall, when considering the period of infancy specifically (0*–*2 years), the responses changed from a more diffuse activation distributed across multiple channels within each inferior frontal and posterior temporal region in the younger time points to more localized activation within a narrower number of channels during the second year of life suggestive of developmental specialisation.

For auditory social selectivity (vocal > non-vocal): (i) responses were present across frontal and temporal regions at 5, 8, 12, 18, 24 months and 3–5 years and (ii) contrary to the visual social selective response, which revealed a narrowing of active channels across 0*–*2 years, auditory social selectivity at 5 and 8 months was found in a smaller number of channels, which became more widespread across inferior frontal, anterior temporal, and posterior temporal regions at 1*–*5 years. Furthermore, (iii) while the 18 months age point was in line with the observed pattern of developmental specialization in the visual social contrast, the auditory social selective response patterns were markedly different to the overall trajectory seen. Finally, the individual longitudinal infant analyses of the sub-sample (N = 50) with valid data at all timepoints over the first year enabled us to group infants based on their age of selectivity towards auditory social stimuli in anterior temporal regions: selectivity emerging at 5 months, 8 months, 12 months, and later than 12 months.

The visual social selectivity confirms previous studies from the UK, the Netherlands, The Gambia and Bangladesh also showing brain activation in the posterior temporal regions in infants from 4 to 6 months of age (Blanco et al., 2023; Braukmann et al., 2018; Frijia et al., 2021; Lloyd-Fox et al., 2009, 2011, 2013, 2017, 2018; Lloyd-Fox, Papademetriou, et al., 2014; Perdue et al., 2019; Pirazzoli et al., 2022; Siddiqui et al., 2023). Furthermore, in line with studies with adults (i.e., Pelphrey et al., 2005) several of these previous studies also find social visual selective patterns of inferior frontal brain activation to similar social stimuli (Blanco et al., 2023; Frijia et al., 2021; Lloyd-Fox et al., 2009, 2011; Perdue et al., 2019; Pirazzoli et al., 2022) confirming that this region of the brain is recruited at this stage of infant development. Evidence of visual social selectivity in the second year of life (Lloyd-Fox et al., 2017; Pirazzoli et al., 2022) and during pre-school years (Perdue et al., 2019; Pirazzoli et al., 2022) is less common. Interestingly, while many of the previous publications in young infancy are from cross-sectional studies conducted in European specialist research labs, previous research that extends longitudinally into the pre-school years (2- to 5-year-olds) has to date only been conducted by the current study and the study from Bangladesh. The current study builds on this evidence by integrating a significantly higher number and density of longitudinal time points into the protocol to better understand the developmental profile across the first 5 years of life. Firstly, this developmental trajectory of brain specialisation to visual social selectivity revealed greater recruitment of inferior frontal brain regions when infants are young (5–8 months) and then returning to this pattern again in late toddlerhood to pre-school age (2–5 years). This later reemergence of frontal responses is in line with previous findings from the Bangladesh cohort (Perdue et al., 2019; Pirazzoli et al., 2022). One possibility that could explain this additional recruitment of frontal regions in pre-schoolers is if the children are viewing the stimuli with more in-depth processing. It is plausible that they could be trying to understand and interpret the intentions or meaning of the actors in the videos in a more complex manner when they reach this age, thus recruiting higher attentional resources in frontal regions. This would align with previous functional magnetic resonance imaging (fMRI) work in adults showing recruitment of frontal cortical regions when interpreting more complex social abilities such as inferring others’ mental states (i.e., Saxe & Wexler, 2005) and interpreting intention in actions (i.e., Saxe et al., 2004). Currently further work is required to better understand social brain network function in the pre-school years as due to the number of challenges of undertaking neuroimaging studies in this age range, this remains an age period that is relatively under-investigated. The additional age points used in our study allowed a deeper understanding of the response profile across the first 2 years. In line with previous fMRI research in adults (Allison et al., 2000; Pelphrey et al., 2005), the posterior temporal area revealed a consistent socially selective response across all six time points. Furthermore, findings revealed that the visual social brain responses specialised from a broad region of temporal and frontal activation at 5–8 months to more restricted clusters within the posterior temporal region by 12–18 months. These findings replicate a previous study (Lloyd-Fox et al., 2017) from different cohorts measured in The Gambia with the same paradigm (though note that only the temporal cortex was measured in this work). One possibility is that regions of the social brain critical for detecting communicative situations specialise rapidly across the first 2 years, reflecting neural interactive specialization (Johnson, 2001). As the individual ages, the brain response becomes more specialized (and less diffuse) within each cortical region as the regions orchestrate a network of connected responses (Johnson et al., 2009). This could help us to understand why 12-18-month-olds did not respond to these stimuli with an activation as widespread as the younger infants.

This study also showed auditory social selective (vocal > non-vocal) brain activation, with particularly consistent activation across all age points in the anterior temporal regions. This concurs with previous findings in fNIRS and fMRI studies at 4 to 7 months (Blasi et al., 2011; Collins-Jones et al., 2024; Grossmann et al., 2010; Lloyd-Fox et al., 2012, 2013, 2017; Minagawa-Kawai et al., 2011; Perdue et al., 2019; Pirazzoli et al., 2022; Shultz et al., 2014), the second year of life (Lloyd-Fox et al., 2017; Pirazzoli et al., 2022), pre-school 3–5 years (Perdue et al., 2019; Pirazzoli et al., 2022) and into adulthood in fMRI work (Belin et al., 2000; Pernet et al., 2015). While auditory social selectivity at 5 and 8 months replicated previous findings of a localised anterior temporal response, the response became more widespread across inferior frontal, anterior temporal and posterior temporal regions at 1–5 years. In the fMRI literature with adults, wide patterns of voice specific activation are also found in the superior temporal cortex (Belin et al., 2000; Frühholz et al., 2020; Pernet et al., 2015). Another previous work describes widespread activation across these brain regions with fNIRS in a previous cohort in Bangladesh. This was observed consistently across two time points at 6 months and 2 years of age, though a different cohort measured at 3 years revealed a very different pattern of responses, with very weak and isolated to temporal regions (Perdue et al., 2019; Pirazzoli et al., 2022). It is possible, given the findings from our work in combination with the published literature, that this indicates that contextual factors can significantly impact individual variance in the developmental profile of this response. It has been noted in previous work that the 4–7 month auditory socially selective responses have considerably varied across studies, sometimes revealing left lateralized responses (Blanco et al., 2023; Lloyd-Fox et al., 2011; Minagawa-Kawai et al., 2011; Shultz et al., 2014), sometimes right lateralised (Lloyd-Fox et al., 2013), sometimes bilateral (Grossmann et al., 2010; Perdue et al., 2019). Furthermore, age related changes even within this small developmental window have been observed, explaining some of the individual variance (Grossmann et al., 2010; Lloyd-Fox et al., 2011). While we are lacking previous evidence for the developmental window from 1–5 years, there are two possible explanations for the differences observed across the current study and previous work. Firstly, it could be driven by methodological factors, which could have impacted the strength of the observed signal as well as the power to observe statistically significant responses. The sample sizes used in the current study from The Gambia and the previously published work from Bangladesh (Perdue et al., 2019; Pirazzoli et al., 2022) ranged from 111 to 155 participants for each age point at which the cohorts were analyzed. Conversely, for the previous cross-sectional studies from The Gambia, UK, Germany, USA., and Japan (Grossmann et al., 2010; Lloyd-Fox et al., 2012, 2013, 2017; Minagawa-Kawai et al., 2011; Shultz et al., 2014) the sample sizes were much lower ranging from 12 to 33 participants. Therefore, the findings for those studies in the Gambia and Bangladesh with the higher samples, providing more statistical power, are likely more robust. While different stimulus contrasts (for example, vocal emotional and non-emotional cues, speech, human, animal), processing methods and thresholding of statistical effects could have driven differences in the localization of the regionally reported responses in some of this previous work, we can be confident that the stimulus presentation, pre-processing steps, statistical methods employed and statistical thresholds set across several of our own publications were very similar and could not explain the differences in activation locations, spread of response or condition differences (Lloyd-Fox et al., 2012, 2017, current study).

Therefore, while methodological considerations could have influenced some of these findings, the age-related differences in the spread of activation into regions beyond the widely reported anterior temporal response are more likely to be explained by contextual factors that could impact an individual’s response. For example, why extensive activation in frontal-temporal regions were seen in the current study from 5 month to 5 years and in one Bangladesh cohort at 6 months and 2 years (whilst the second Bangladesh cohort measured at 3 years reports a very isolated small temporal response). Of interest, we observed a sharp decrease in the spread of activation at 18 months, with only 3 bilateral temporal channels showing activation on the contrast versus 27 at 12 months and 23 at 24 months, which we cannot attribute to lower data quality at this age. Indeed, the anterior temporal location of the auditory socially selective response at 18 months aligns with the most consistent response at all other ages as well as with previous work. This is very similar to the Bangladesh study where only 3 isolated bilateral channels showed activation at 3 years (Pirazzoli et al., 2022), also within these regions. While the response in the Bangladesh work comes from a distinct cohort and may represent individual variance in group level responses, it is more difficult to explain in our longitudinal study. Additional studies would need to be conducted in order to investigate the unexpected auditory social selectivity findings at 18 months.

It is possible that insights gained from the individual participant neural profiles will help us understand the level of individual variance. Previous work has shown that 0–9 months is a period of transition, where some infants may respond more strongly to non-vocal stimuli while others to vocal stimuli (i.e., Blanco et al., 2023; Lloyd-Fox et al., 2012). In the current study, we identified the age at which infants started showing a greater anterior temporal activation in response to *auditory social (vocal)* compared to *auditory non-social (non-vocal)* stimuli. The proportion of infants already vocal selective at 5 months on the restricted sample with all sessions from 5 to 12 months (50 participants) was 52%, which is close to what we see on the larger sample with the 127 participants with valid cross-sectional data at the 5 months session (55 %). Furthermore, two thirds of these infants continued to show consistent vocal selectivity at later age points. This, however, did not mean that their selectivity was already significant, as we were not able to test this statistically at the individual level due to the limited number of trials per condition for each participant (as low as 3 for some infants). Nonetheless, we believe it was still useful to build such group-based thresholds of participants’ block-averaged contrast responses, as it enabled us to have an indication of their specialisation trend. The age of selectivity in our study did appear to be in accordance with previous literature, highlighting variance in vocal selectivity at 4 to 8 months (Grossmann et al., 2010; Lloyd-Fox et al., 2012), which becomes more consistent at later ages. It seemed established, however, that while a temporal auditory response is detected during the first week of life the development of selective social perception at this age is not yet mature, as shown by Cristia et al. (2014), and reveals considerable individual variation (see Greenhalgh et al., 2025 for analyses at 1 months from the BRIGHT project). While the significant positive effect of age on the probability of auditory social selectivity suggests in our study that older participants are more likely to show social selectivity, the substantial variability in the random intercepts indicates that there are individual differences that are not captured by the fixed effects alone. This highlights the importance of building different profiles of social specialisation, which supports our proposal of an approach to group participants based on the age of first social selectivity, showing different developmental trajectories for brain responses to social and non-social stimuli. Those different social specialisation profiles (subgroups) will become useful for future work to see if they could be linked to adversity or resilience factors. In the Bangladesh study, differences in activation patterns for both the visual social selective responses and the auditory selective responses were partially explained by age dependent associations with contextual factors. Cortical social discrimination at 2 years related to family reported intimate partner violence, verbal abuse and family conflict, while at 3 years it related to verbal abuse and family conflict and maternal depression (Pirazzoli et al., 2022). No associations were found with the younger age point of 6 months. In future work we aim to model the current findings with broader contextual factors prevalent in the rural population where the BRIGHT Project has taken place (Lloyd-Fox et al., 2024) to explore the impact of both poverty associate risk and enriched family contexts.

### 4.2 Methodological consideration

Studying HRF characteristics was very important here, as we saw that both the time-to-peak and magnitude of the HRFs were affected by the experimental condition and age. It enabled us to use adaptive delays for the time windows used for signal averaging, which prevented introducing bias towards an age, a participant, a brain region, or a condition.

The investigations of those HRF characteristics led us to conclude first of all that because the time-to-peak was influenced by the stimuli condition (Fig. 5), it was important to select a time window for feature extraction that was centered around the average time-to-peak across the compared conditions. Secondly, the fact that the time-to-peak was influenced by the age of participant (Fig. 5) added confidence that the time-to-peak selected for window centring should also be age dependent and therefore calculated separately for every age point. Finally, the fact that age was also influencing the magnitudes of the responses (Fig. 6), combined with the growing head circumference throughout infancy (Table 1) led us to forgo using HRF amplitude features directly for any comparisons across age points. Instead, we focused on the sign of the contrast (selectivity), rather than comparing values directly. While the differences in maximum amplitude cannot all be attributed to neural correlates alone and could originate, for example from differences in skull thickness (Li et al., 2015), it remains nonetheless very important to keep this in mind, especially when comparing results at different age points. This is why for the longitudinal analysis, the focus was on relative changes between conditions (social or non-social selectivity) and age of first social selectivity, rather than HbO or HbR amplitude values.

Selecting a time-window for extracting and HRF average amplitude is a very common practice in fNIRS literature. This is especially the case for studies with infants because other approaches such as general linear model (GLM) are harder to use with infant populations (Filippetti et al., 2023). While a fixed window selection could work when doing cross-sectional studies (Gemignani & Gervain, 2021), we showed here that it was very important to consider adaptive latencies when stimuli are different and the study is longitudinal.

### 4.3 Recommendations for individual differences analyses for future work

Based on our work, we propose the following recommendations:

1. We recommend exploring the anatomical spread of the response across the network of regions and not just the localisation of the peak response as this can reveal important age- or stimulus-related differences.
2. We also recommend exploring the latency of the response to different stimuli and at different ages to ensure the use of an analytical approach that can accommodate age- and stimulus- related changes that may be theoretically important.
3. Finally, future research should focus on using multiple longitudinal time points to understand not only if the responses are appropriate for a given age point, but also if deviations from a trajectory at the individual level are transient or related to a change in cognitive processing that is theoretically important.

## 5 Conclusions

This study contributes to the understanding of the development of social perception in infancy by examining brain responses to *auditory social (vocal)*, *auditory non-social (non-vocal)* and *visual social (silent)* stimuli longitudinally from 5 months to 3–5 years of age. Our findings indicate infants have a specialised neural response towards social stimuli, even as early as 5 months of age. Specifically, we observed a consistent posterior temporal selectivity for social visual stimuli across all ages, with some changes in inferior frontal regions, showing selectivity in the youngest and oldest age groups. Social vocal stimuli elicited a widespread and sustained response across temporal areas in this longitudinal cohort, extending to inferior frontal areas from 12 months. Moreover, our longitudinal analysis revealed distinct developmental trajectories in individual infants’ specialization to social vocal stimuli, with infants generally remaining social selective after they showed their first social specialisation.

These results highlight the complexity of the developmental changes in the neural processing of auditory social information during infancy and early childhood and open to future research to investigate how these trajectories may be influenced by environmental factors (adversity or resilience factors) and how they relate to later social and cognitive outcomes. The methodology developed in this study, which combines the analysis of the latency and magnitude of the hemodynamic response with the characterisation of individual developmental profiles, can be valuable for future studies in developmental cognitive neuroscience, especially in under-represented populations and in low-resource settings.

## Data Availability

As described in detail in the BRIGHT Project publication describing the full study protocol (Lloyd-Fox et al., 2024), access to any data collected during or generated by the BRIGHT Project is fully audited, and to ensure data security, is overseen by the data management team in the UK and The Gambia. While data sharing is critically important to maximising the benefit of research, the BRIGHT Project must also consider the need to protect the confidentiality of this sensitive group (particularly the infants within the mother-infant dyads, who as minors do not consent for themselves). The dataset is unique in that it provides the ability to link data points together (i.e. fNIRS data with outcome data or contextual factor data), but due to the nature of the data being collected (i.e., collected from a specific geographical location, longitudinal dataset of several datapoints) the majority of the data cannot be fully de-identified under the guidance included in the European General Data Protection Regulation (GDPR), and the conditions of our ethics approval do not allow public archiving of pseudonymised study data. Indeed, while fNIRS recordings can be anonymised, some of the demographic information used in this work cannot be deidentified. However, collaborations are encouraged, and projects are evaluated primarily on their consistency with the ethical principles and aims of the project that the families signed up to when partaking in this study. To access the data, interested readers should contact the BRIGHT coordinator on our website (https://www.globalfnirs.org/contact). Access will be granted to named individuals following ethical procedures governing the reuse of sensitive data. Requestors must complete and sign a data sharing agreement to ensure data is stored securely and used in accordance with the BRIGHT Project’s policies on Ethics, Data Sharing, Authorship, and Publication. The data analysis code is available on GitHub (https://github.com/globalfnirs).

## References

[IMAG.a.1368-b1] Allison, T., Puce, A., & McCarthy, G. (2000). Social perception from visual cues: Role of the STS region. Trends in Cognitive Sciences, 4(7), 267–278. 10.1016/s1364-6613(00)01501-1 10859571

[IMAG.a.1368-b2] Azhari, A., Truzzi, A., Neoh, M. J., Balagtas, J. P. M., Tan, H. H., Goh, P. P., Ang, X. A., Setoh, P., Rigo, P., Bornstein, M. H., & Esposito, G. (2020). A decade of infant neuroimaging research: What have we learned and where are we going? Infant Behavior & Development, 58, 101389. 10.1016/j.infbeh.2019.101389 31778859

[IMAG.a.1368-b3] Belin, P., Zatorre, R. J., Lafaille, P., Ahad, P., & Pike, B. (2000). Voice-selective areas in human auditory cortex. Nature, 403(6767), 309–312. 10.1038/35002078 10659849

[IMAG.a.1368-b4] Benjamini, Y., & Hochberg, Y. (1995). Controlling the false discovery rate: A practical and powerful approach to multiple testing. Journal of the Royal Statistical Society: Series B (Methodological), 57(1), 289–300. 10.1111/j.2517-6161.1995.tb02031.x

[IMAG.a.1368-b5] Blanco, B., Lloyd-Fox, S., Begum-Ali, J., Pirazzoli, L., Goodwin, A., Mason, L., Pasco, G., Charman, T., Jones, E. J. H., Johnson, M. H., & BASIS/STAARS Team. (2023). Cortical responses to social stimuli in infants at elevated likelihood of ASD and/or ADHD: A prospective cross-condition fNIRS study. Cortex, 169, 18–34. 10.1016/j.cortex.2023.07.010 37847979

[IMAG.a.1368-b6] Blasi, A., Lloyd-Fox, S., Johnson, M. H., & Elwell, C. (2014). Test–retest reliability of functional near infrared spectroscopy in infants. Neurophotonics, 1(2), 025005–5. 10.1117/1.nph.1.2.025005 PMC4478781 26157978

[IMAG.a.1368-b7] Blasi, A., Lloyd-Fox, S., Katus, L., & Elwell, C. E. (2019). fNIRS for tracking brain development in the context of global health projects. Photonics, 6(3), 89. 10.3390/photonics6030089 PMC7745110 33490142

[IMAG.a.1368-b8] Blasi, A., Mercure, E., Lloyd-Fox, S., Thomson, A., Brammer, M., Sauter, D., Deeley, Q., Barker, G. J., Renvall, V., Deoni, S., Gasston, D., Williams, S. C., Johnson, M. H., Simmons, A., & Murphy, D. G. (2011). Early specialization for voice and emotion processing in the infant brain. Current Biology, 21(14), 1220–1224. 10.1016/j.cub.2011.06.009 21723130

[IMAG.a.1368-b9] Braukmann, R., Lloyd-Fox, S., Blasi, A., Johnson, M. H., Bekkering, H., Buitelaar, J. K., & Hunnius, S. (2018). Diminished socially selective neural processing in 5‐month‐old infants at high familial risk of autism. European Journal of Neuroscience, 47(6), 720–728. 10.1111/ejn.13751 PMC5943701 29057566

[IMAG.a.1368-b10] Bulgarelli, C., Blasi, A., Pollonini, L., Lloyd-Fox, S., Pirazzoli, L., Perdue, K. L., Nelson, C. A., & Elwell, C. E. (2020). Standardising an infant fNIRS analysis pipeline to investigate neurodevelopment in global health. In Optics and the Brain (Paper BM2C–2). Optica Publishing Group. 10.1364/brain.2020.bm2c.2

[IMAG.a.1368-b11] Collins-Jones, L. H., Cooper, R. J., Bulgarelli, C., Blasi, A., Katus, L., McCann, S., Mason, L., Mbye, E., Touray, E., Ceesay, M., Moore, S. E., Lloyd-Fox, S., Elwell, C. E., & BRIGHT Study Team. (2021). Longitudinal infant fNIRS channel-space analyses are robust to variability parameters at the group-level: An image reconstruction investigation. NeuroImage, 237, 118068. 10.1016/j.neuroimage.2021.118068 PMC8285580 33915275

[IMAG.a.1368-b12] Collins-Jones, L. H., Gossé, L. K., Blanco, B., Bulgarelli, C., Siddiqui, M., Vidal-Rosas, E. E., Duobaitė, N., Nixon-Hill, R. W., Smith, G., Skipper, J., Sargent, T., Powell, S., Everdell, N. L., Jones, E. J. H., & Cooper, R. J. (2024). Whole-head high-density diffuse optical tomography to map infant audio-visual responses to social and non-social stimuli. Imaging Neuroscience, 2, 1–19. 10.1162/imag_a_00244 PMC12272203 40800512

[IMAG.a.1368-b13] Cristia, A., Dupoux, E., Hakuno, Y., Lloyd-Fox, S., Schuetze, M., Kivits, J., Bergvelt, T., van Gelder, M., Filippin, L., Charron, S., & Minagawa-Kawai, Y. (2013). An online database of infant functional near infrared spectroscopy studies: A community-augmented systematic review. PLoS One, 8(3), e58906. 10.1371/journal.pone.0058906 PMC3598807 23554955

[IMAG.a.1368-b14] Cristia, A., Minagawa, Y., & Dupoux, E. (2014). Responses to vocalizations and auditory controls in the human newborn brain. PLoS One, 9(12), e115162. 10.1371/journal.pone.0115162 PMC4269432 25517997

[IMAG.a.1368-b15] Delpy, D. T., Cope, M., van der Zee, P., Arridge, S. R., Wray, S., & Wyatt, J. S. (1988). Estimation of optical pathlength through tissue from direct time of flight measurement. Physics in Medicine & Biology, 33(12), 1433. 10.1088/0031-9155/33/12/008 3237772

[IMAG.a.1368-b16] Di Lorenzo, R., Pirazzoli, L., Blasi, A., Bulgarelli, C., Hakuno, Y., Minagawa, Y., & Brigadoi, S. (2019). Recommendations for motion correction of infant fNIRS data applicable to multiple data sets and acquisition systems. Neuroimage, 200, 511–527. 10.1016/j.neuroimage.2019.06.056 31247300

[IMAG.a.1368-b17] Elder, G. H. (1979). Historical change in life patterns and personality. Life-Span Development and Behavior, 2, 117–159. 10.4324/9781315789255-4

[IMAG.a.1368-b18] Everdell, N. L., Gibson, A. P., Tullis, I. D. C., Vaithianathan, T., Hebden, J. C., & Delpy, D. T. (2005). A frequency multiplexed near-infrared topography system for imaging functional activation in the brain. Review of Scientific Instruments, 76(9), 093705. 10.1063/1.2038567

[IMAG.a.1368-b19] Filippetti, M. L., Andreu-Perez, J., de Klerk, C., Richmond, C., & Rigato, S. (2023). Are advanced methods necessary to improve infant fNIRS data analysis? An assessment of baseline-corrected averaging, general linear model (GLM) and multivariate pattern analysis (MVPA) based approaches. NeuroImage, 265, 119756. 10.1016/j.neuroimage.2022.119756

[IMAG.a.1368-b20] Frijia, E. M., Billing, A., Lloyd-Fox, S., Vidal Rosas, E., Collins-Jones, L., Crespo-Llado, M. M., Amadó, M. P., Austin, T., Edwards, A., Dunne, L., Smith, G., Nixon-Hill, R., Powell, S., Everdell, N. L., & Cooper, R. J. (2021). Functional imaging of the developing brain with wearable high-density diffuse optical tomography: A new benchmark for infant neuroimaging outside the scanner environment. NeuroImage, 225, 117490. 10.1016/j.neuroimage.2020.117490 33157266

[IMAG.a.1368-b21] Frühholz, S., Trost, W., Grandjean, D., & Belin, P. (2020). Neural oscillations in human auditory cortex revealed by fast fMRI during auditory perception. NeuroImage, 207, 116401. 10.1016/j.neuroimage.2019.116401 31783116

[IMAG.a.1368-b22] Gemignani, J., & Gervain, J. (2021). Comparing different pre-processing routines for infant fNIRS data. Developmental Cognitive Neuroscience, 48, 100943. 10.1016/j.dcn.2021.100943 PMC7985709 33735718

[IMAG.a.1368-b23] Gervain, J., Minagawa, Y., Emberson, L., & Lloyd-Fox, S. (2023). Using functional near-infrared spectroscopy to study the early developing brain: Future directions and new challenges. Neurophotonics, 10(2), 023519–023519. 10.1117/1.nph.10.2.023519 PMC10068680 37020727

[IMAG.a.1368-b24] Gilmore, J. H., Knickmeyer, R. C., & Gao, W. (2018). Imaging structural and functional brain development in early childhood. Nature Reviews Neuroscience, 19(3), 123–137. 10.1038/nrn.2018.1 PMC5987539 29449712

[IMAG.a.1368-b25] Grantham-McGregor, S. M., Powell, C. A., Walker, S. P., & Himes, J. H. (1991). Nutritional supplementation, psychosocial stimulation, and mental development of stunted children: The Jamaican Study. The Lancet, 338(8758), 1–5. 10.1016/0140-6736(91)90001-6 1676083

[IMAG.a.1368-b26] Greenhalgh, I., Blanco, B., Bulgarelli, C., Mbye, E., Touray, E., Rozhko, M., Katus, L., Hayes, N., McCann, S., Moore, S. E., Elwell, C., Blasi, A., Lloyd-Fox, S., & the BRIGHT Project Team. (2025). Cross-paradigm fNIRS brain activity in neonates across the Gambia and UK. BioRxiv. 10.1101/2025.07.28.666710 PMC12831619 41585349

[IMAG.a.1368-b27] Grossmann, T., Oberecker, R., Koch, S. P., & Friederici, A. D. (2010). The developmental origins of voice processing in the human brain. Neuron, 65(6), 852–858. 10.1016/j.neuron.2010.03.001 PMC2852650 20346760

[IMAG.a.1368-b28] Hennig, B. J., Unger, S. A., Dondeh, B. L., Hassan, J., Hawkesworth, S., Jarjou, L., Jones, K. S., Moore, S. E., Nabwera, H. M., Ngum, M., Prentice, A., Sonko, B., Prentice, A. M., & Fulford, A. J. (2017). Cohort profile: The Kiang West Longitudinal Population Study (KWLPS)-A platform for integrated research and health care provision in rural Gambia. International Journal of Epidemiology, 46(2), e13. 10.1093/ije/dyv206 PMC5837564 26559544

[IMAG.a.1368-b29] Hernandez, S. M., & Pollonini, L. (2020). NIRSplot: A tool for quality assessment of fNIRS scans. In Optics and the Brain (Paper BM2C–5). Optica Publishing Group. 10.1364/brain.2020.bm2c.5

[IMAG.a.1368-b30] Huppert, T. J., Diamond, S. G., Franceschini, M. A., & Boas, D. A. (2009). HomER: A review of time-series analysis methods for near-infrared spectroscopy of the brain. Applied Optics, 48(10), D280–D298. 10.1364/ao.48.00d280 PMC2761652 19340120

[IMAG.a.1368-b31] Jensen, S. K. G., Berens, A. E., & Nelson, C. A. (2017). Effects of poverty on interacting biological systems underlying child development. The Lancet Child & Adolescent Health, 1(3), 225–239. 10.1016/s2352-4642(17)30024-x 30169171

[IMAG.a.1368-b32] Jensen, S. K. G., Kumar, S., Xie, W., Tofail, F., Haque, R., Petri, W. A., & Nelson, C. A. (2019). Neural correlates of early adversity among Bangladeshi infants. Scientific Reports, 9(1), 3507. 10.1038/s41598-019-39242-x PMC6401115 30837491

[IMAG.a.1368-b33] Johnson, M. H. (2001). Functional brain development in humans. Nature Reviews Neuroscience, 2(7), 475–483. 10.1038/35081509 11433372

[IMAG.a.1368-b34] Johnson, M. H., Grossmann, T., & Cohen Kadosh, K. (2009). Mapping functional brain development: Building a social brain through interactive specialization. Developmental Psychology, 45(1), 151. 10.1037/a0014548 19209998

[IMAG.a.1368-b35] Li, Z., Park, B.-K., Liu, W., Zhang, J., Reed, M. P., Rupp, J. D., Hoff, C. N., & Hu, J. (2015). A statistical skull geometry model for children 0-3 years old. PLoS One, 10(5), e0127322. 10.1371/journal.pone.0127322 PMC4436309 25992998

[IMAG.a.1368-b36] Lloyd-Fox, S., Begus, K., Halliday, D., Pirazzoli, L., Blasi, A., Papademetriou, M., Darboe, M. K., Prentice, A. M., Johnson, M. H., Moore, S. E., & Elwell, C. E. (2017). Cortical specialisation to social stimuli from the first days to the second year of life: A rural Gambian cohort. Developmental Cognitive Neuroscience, 25, 92–104. 10.1016/j.dcn.2016.11.005 PMC5485636 28017265

[IMAG.a.1368-b37] Lloyd-Fox, S., Blasi, A., Elwell, C. E., Charman, T., Murphy, D., & Johnson, M. H. (2013). Reduced neural sensitivity to social stimuli in infants at risk for autism. Proceedings of the Royal Society B: Biological Sciences, 280(1758), 20123026. 10.1098/rspb.2012.3026 PMC3619456 23486434

[IMAG.a.1368-b38] Lloyd-Fox, S., Blasi, A., Everdell, N., Elwell, C. E., & Johnson, M. H. (2011). Selective cortical mapping of biological motion processing in young infants. Journal of Cognitive Neuroscience, 23(9), 2521–2532. 10.1162/jocn.2010.21598 20954934

[IMAG.a.1368-b39] Lloyd-Fox, S., Blasi, A., Mercure, E., Elwell, C. E., & Johnson, M. H. (2012). The emergence of cerebral specialization for the human voice over the first months of life. Social Neuroscience, 7(3), 317–330. 10.1080/17470919.2011.614696 21950945

[IMAG.a.1368-b40] Lloyd-Fox, S., Blasi, A., Pasco, G., Gliga, T., Jones, E. J. H., Murphy, D. G. M., Elwell, C. E., Charman, T., Johnson, M. H., & BASIS Team. (2018). Cortical responses before 6 months of life associate with later autism. The European Journal of Neuroscience, 47(6), 736–749. 10.1111/ejn.13757 PMC5900943 29057543

[IMAG.a.1368-b41] Lloyd-Fox, S., Blasi, A., Volein, A., Everdell, N., Elwell, C. E., & Johnson, M. H. (2009). Social perception in infancy: A near infrared spectroscopy study. Child Development, 80(4), 986–999. 10.1111/j.1467-8624.2009.01312.x 19630889

[IMAG.a.1368-b42] Lloyd-Fox, S., McCann, S., Milosavljevic, B., Katus, L., Blasi, A., Bulgarelli, C., Crespo-Llado, M., Ghillia, G., Fadera, T., Mbye, E., Mason, L., Njai, F., Njie, O., Perapoch-Amado, M., Rozhko, M., Sosseh, F., Saidykhan, M., Touray, E., Moore, S. E., Elwell, C. E., … BRIGHT Project Team. (2024). The Brain Imaging for Global Health (BRIGHT) Project: Longitudinal cohort study protocol. Gates Open Research, 7, 126. 10.12688/gatesopenres.14795.2 PMC11452580 39372355

[IMAG.a.1368-b43] Lloyd-Fox, S., Papademetriou, M., Darboe, M. K., Everdell, N. L., Wegmuller, R., Prentice, A. M., Moore, S. E., & Elwell, C. E. (2014). Functional near infrared spectroscopy (fNIRS) to assess cognitive function in infants in rural Africa. Scientific Reports, 4(1), 4740. 10.1038/srep04740 PMC5381189 24751935

[IMAG.a.1368-b44] Lloyd-Fox, S., Richards, J. E., Blasi, A., Murphy, D. G. M., Elwell, C. E., & Johnson, M. H. (2014). Coregistering functional near-infrared spectroscopy with underlying cortical areas in infants. Neurophotonics, 1(2), 025006–6. 10.1117/1.nph.1.2.025006 PMC4280679 25558463

[IMAG.a.1368-b45] Luke, R., Larson, E., Shader, M. J., Innes-Brown, H., Van Yper, L., Lee, A. K. C., Sowman, P. F., & McAlpine, D. (2021). Analysis methods for measuring passive auditory fNIRS responses generated by a block-design paradigm. Neurophotonics, 8(2), 025008–025008. 10.1117/1.nph.8.2.025008 PMC8140612 34036117

[IMAG.a.1368-b46] McCoy, D. C., Peet, E. D., Ezzati, M., Danaei, G., Black, M. M., Sudfeld, C. R., Fawzi, W., & Fink, G. (2016). Early childhood developmental status in low-and middle-income countries: National, regional, and global prevalence estimates using predictive modeling. PLoS Medicine, 13(6), e1002034. 10.1371/journal.pmed.1002034 PMC4896459 27270467

[IMAG.a.1368-b47] Minagawa-Kawai, Y., Van Der Lely, H., Ramus, F., Sato, Y., Mazuka, R., & Dupoux, E. (2011). Optical brain imaging reveals general auditory and language-specific processing in early infant development. Cerebral Cortex, 21(2), 254–261. 10.1093/cercor/bhq082 PMC3020578 20497946

[IMAG.a.1368-b48] Nabwera, H. M., Fulford, A. J., Moore, S. E., & Prentice, A. M. (2017). Growth faltering in rural Gambian children after four decades of interventions: A retrospective cohort study. The Lancet Global Health, 5(2), e208–e216. 10.1016/s2214-109x(16)30355-2 PMC5340725 28104187

[IMAG.a.1368-b49] Pelphrey, K. A., Morris, J. P., Michelich, C. R., Allison, T., & McCarthy, G. (2005). Functional anatomy of biological motion perception in posterior temporal cortex: An fMRI study of eye, mouth and hand movements. Cerebral Cortex, 15(12), 1866–1876. 10.1093/cercor/bhi064 15746001

[IMAG.a.1368-b50] Perdue, K. L., Jensen, S. K. G., Kumar, S., Richards, J. E., Kakon, S. H., Haque, R., Petri Jr, W. A., Lloyd-Fox, S., Elwell, C., & Nelson, C. A. (2019). Using functional near-infrared spectroscopy to assess social information processing in poor urban Bangladeshi infants and toddlers. Developmental Science, 22(5), e12839. 10.1111/desc.12839 PMC6737924 31017372

[IMAG.a.1368-b51] Pernet, C. R., McAleer, P., Latinus, M., Gorgolewski, K. J., Charest, I., Bestelmeyer, P. E., Watson, R. H., Fleming, D., Crabbe, F., Valdes-Sosa, M., & Belin, P. (2015). The human voice areas: Spatial organization and inter-individual variability in temporal and extra-temporal cortices. NeuroImage, 119, 164–174. 10.1016/j.neuroimage.2015.06.050 PMC4768083 26116964

[IMAG.a.1368-b52] Pirazzoli, L., Sullivan, E., Xie, W., Richards, J. E., Bulgarelli, C., Lloyd-Fox, S., Shama, T., Kakon, S. H., Haque, R., Petri, W. A. J., & Nelson, C. A., 3rd (2022). Association of psychosocial adversity and social information processing in children raised in a low-resource setting: an fNIRS study. Developmental Cognitive Neuroscience, 56, 101125. 10.1016/j.dcn.2022.101125 PMC9241055 35763916

[IMAG.a.1368-b53] Pollonini, L., Olds, C., Abaya, H., Bortfeld, H., Beauchamp, M. S., & Oghalai, J. S. (2014). Auditory cortex activation to natural speech and simulated cochlear implant speech measured with functional near-infrared spectroscopy. Hearing Research, 309, 84–93. 10.1016/j.heares.2013.11.007 PMC3939048 24342740

[IMAG.a.1368-b54] Saxe, R., & Wexler, A. (2005). Making sense of another mind: The role of the right temporo-parietal junction. Neuropsychologia, 43(10), 1391–1399. 10.1016/j.neuropsychologia.2005.02.013 15936784

[IMAG.a.1368-b55] Saxe, R., Xiao, D. K., Kovacs, G., Perrett, D. I., & Kanwisher, N. (2004). A region of right posterior superior temporal sulcus responds to observed intentional actions. Neuropsychologia, 42(11), 1435–1446. 10.1016/j.neuropsychologia.2004.04.015 15246282

[IMAG.a.1368-b56] Schoenbuchner, S. M., Dolan, C., Mwangome, M., Hall, A., Richard, S. A., Wells, J. C., Khara, T., Sonko, B., Prentice, A. M., & Moore, S. E. (2019). The relationship between wasting and stunting: A retrospective cohort analysis of longitudinal data in Gambian children from 1976 to 2016. The American Journal of Clinical Nutrition, 110(2), 498–507. 10.1093/ajcn/nqy326 PMC6669055 30753251

[IMAG.a.1368-b57] Seabold, S., & Perktold, J. (2010). Statsmodels: Econometric and statistical modeling with Python. In 9th Python in Science Conference. 10.25080/majora-92bf1922-011

[IMAG.a.1368-b58] Shultz, S., Vouloumanos, A., Bennett, R. H., & Pelphrey, K. (2014). Neural specialization for speech in the first months of life. Developmental Science, 17(5), 766–774. 10.1111/desc.12151 PMC4232861 24576182

[IMAG.a.1368-b59] Siddiqui, M., Pinti, P., Brigadoi, S., Lloyd-Fox, S., Elwell, C. E., Johnson, M. H., Tachtsidis, I., & Jones, E. J. H. (2023). Using multi-modal neuroimaging to characterise social brain specialisation in infants. Elife, 12, e84122. 10.7554/elife.84122 PMC10624424 37818944

[IMAG.a.1368-b60] Strangman, G., Culver, J. P., Thompson, J. H., & Boas, D. A. (2002). A quantitative comparison of simultaneous BOLD fMRI and NIRS recordings during functional brain activation. Neuroimage, 17(2), 719–731. 10.1006/nimg.2002.1227 12377147

[IMAG.a.1368-b61] Virtanen, P., Gommers, R., Oliphant, T. E., Haberland, M., Reddy, T., Cournapeau, D., Burovski, E., Peterson, P., Weckesser, W., Bright, J., van der Walt, S. J., Brett, M., Wilson, J., Millman, K. J., Mayorov, N., Nelson, A. R. J., Jones, E., Kern, R., Larson, E., Carey, C. J., … SciPy 1.0 Contributors. (2020). SciPy 1.0: Fundamental algorithms for scientific computing in Python. Nature Methods, 17(3), 261–272. 10.1038/s41592-020-0772-5 PMC7056644 32015543

[IMAG.a.1368-b62] Walker, S. P., Chang, S. M., Wright, A. S., Pinto, R., Heckman, J. J., & Grantham‐McGregor, S. M. (2022). Cognitive, psychosocial, and behaviour gains at age 31 years from the Jamaica early childhood stimulation trial. Journal of Child Psychology and Psychiatry, 63(6), 626–635. 10.1111/jcpp.13499 PMC8850528 34403137

[IMAG.a.1368-b63] Wijeakumar, S., Kumar, A., Delgado Reyes, L. M., Tiwari, M., & Spencer, J. P. (2019). Early adversity in rural India impacts the brain networks underlying visual working memory. Developmental Science, 22(5), e12822. 10.1111/desc.12822 PMC6767418 30803122

